# Gamma radiation coupled ADP-ribosyl transferase activity of *Pseudomonas aeruginosa* PE24 moiety

**DOI:** 10.1007/s00253-023-12401-x

**Published:** 2023-02-18

**Authors:** Radwa N. Morgan, Sarra E. Saleh, Hala A. Farrag, Khaled M. Aboshanab

**Affiliations:** 1 National Centre for Radiation Research and Technology (NCRRT), Drug Radiation Research Department, Egyptian Atomic Energy Authority (EAEA), Ahmed El-Zomor Street, Nasr City, 11787 Cairo Egypt; 2 Microbiology and Immunology Department, Faculty of Pharmacy, Ain Shams University, African Union Organization Street, Abbassia, 11566 Cairo Egypt

**Keywords:** *P. aeruginosa* PE24 moiety, ADP-ribosylation, Nitrobenzylidene aminoguanidine (NBAG), Gamma irradiation, Combination therapy

## Abstract

**Abstract:**

The ADP-ribosyl transferase activity of *P. aeruginosa* PE24 moiety expressed by *E. coli* BL21 (DE3) was assessed on nitrobenzylidene aminoguanidine (NBAG) and in vitro cultured cancer cell lines. Gene encoding PE24 was isolated from *P. aeruginosa* isolates, cloned into pET22b( +) plasmid, and expressed in *E. coli* BL21 (DE3) under IPTG induction. Genetic recombination was confirmed by colony PCR, the appearance of insert post digestion of engineered construct, and protein electrophoresis using sodium dodecyl-sulfate polyacrylamide gel (SDS-PAGE). The chemical compound NBAG has been used to confirm PE24 extract ADP-ribosyl transferase action through UV spectroscopy, FTIR, c^13^-NMR, and HPLC before and after low-dose gamma irradiation (5, 10, 15, 24 Gy). The cytotoxicity of PE24 extract alone and in combination with paclitaxel and low-dose gamma radiation (both 5 Gy and one shot 24 Gy) was assessed on adherent cell lines HEPG2, MCF-7, A375, OEC, and Kasumi-1 cell suspension. Expressed PE24 moiety ADP-ribosylated NBAG as revealed by structural changes depicted by FTIR and NMR, and the surge of new peaks at different retention times from NBAG in HPLC chromatograms. Irradiating recombinant PE24 moiety was associated with a reduction in ADP-ribosylating activity. The PE24 extract IC50 values were < 10 μg/ml with an acceptable *R*^2^ value on cancer cell lines and acceptable cell viability at 10 μg/ml on normal OEC. Overall, the synergistic effects were observed upon combining PE24 extract with low-dose paclitaxel demonstrated by the reduction in IC50 whereas antagonistic effects and a rise in IC50 values were recorded after irradiation by low-dose gamma rays.

**Key points:**

• *Recombinant PE24 moiety was successfully expressed and biochemically analyzed*.

• *Low-dose gamma radiation and metal ions decreased the recombinant PE24 cytotoxic activity*.

• *Synergism was observed upon combining recombinant PE24 with low-dose paclitaxel*.

**Supplementary information:**

The online version contains supplementary material available at 10.1007/s00253-023-12401-x.

## Introduction

Several PE-based immunotoxins (PE-IT) were constructed to target several carcinomas for their eminent cytotoxicity, high efficacies, and tolerability (Shafiee et al. 2019; Leshem and Pastan 2019; Havaei et al. 2021). Recent studies utilized PE24 moiety during PE-ITs fabrication where both domains I and II were eliminated. Elimination of both domains was associated with high response rates and reduced levels of vascular leak syndromes in vivo animal models (Wu and Zhu 2021). The PE24 moiety was utilized in SS1-Fab-DS3-PE24 IT, targeting mesothelin expressed by pancreatic carcinoma, mesothelioma gastric, and lung carcinomas (Kaplan et al. 2016). It was also incorporated in anti-B cell maturation antigen (BCMA) disulfide-linked Fv portion containing IT-termed LMB-70 and LMB-75 ITs targeting myeloma cells (Michalska et al. 2018). Single-domain antibody (SdAb) targeting prostate-specific membrane antigen (PSMA) was fused to a genetically modified PE24 moiety termed PE24X7 and exhibited few non-specific toxicities and better effects in an animal model (Xing et al. 2021). Recently, Jun et al. formulated different anti-epidermal growth factor receptor (EGFR) IT harboring an engineered PE24 moiety and tested them in tumor xenograft mouse models exhibiting EGFR receptors. The anti-EGFR PE24-based ITs showed tolerable effects (Jun et al. 2022).

Interestingly, PE-IT therapy combined with chemotherapeutic agents and radiotherapy was associated with favorable outcomes. Hassan et al. noted that the efficacy of SS1P PE-IT targeting mesothelioma was enhanced after irradiation by sublethal radiation dosages and attributed this effect to the upregulation of mesothelin expression (Hassan et al. 2006; Dieffenbach and Pastan 2020). The efficacy of SS1P PE-IT was also enhanced after concurrent administration with tubulin-targeting chemotherapeutic paclitaxel (Taxol) due to the reduction of antigen shedding and enhanced IT toxin uptake by cells (Zhang et al. 2007; Dieffenbach and Pastan 2020). All previously mentioned studies successfully generated different formulations of PE24/PE-based IT and were focused on reducing undesirable toxicities associated with the usage of IT while attaining acceptable anticancer activities. Only a few studies were focused on understanding the effect of different recombination techniques and combination therapies on ADP-ribosyl transferase activity of expressed PE24 moiety. For instance, Boland et al. produced catalytically in active peptide fragments upon the genetic splitting of the PE catalytic domain and recombinant production. These peptide fragments failed to exert an ADP-ribosyl transferase activity on eukaryotic elongation factor 2 and suggested a complementation technique to maintain ADP-ribosyl transferase activity and facilitate recombination with targeting moieties (Boland et al. 2014). This necessitates the testing of the ADP-ribosylating capability of expressed PE24 prior to conjugation to the targeting moiety. Combination with chemotherapeutic agents might reduce the enzymatic activity of PE24 as these agents might compete with NAD^+^ on its active site. Armstrong et al. denoted that the active site of the PE catalytic domain has a high affinity to naphthalimide compounds which exhibited promising anti-tumor effects against breast carcinoma (Armstrong et al. 2002; Lv and Xu 2009). The presence of amide group (found in paclitaxel), bi or tricyclic lactam rings also showed high affinity to the active site of PE24 moiety and was associated with eminent inhibitory effects (Yates et al. 2005; Turunen et al. 2008). In a similar manner, the impact of combining gamma radiation with PE-based IT was assessed in the terms of reducing cancer cell resistance against IT (Dieffenbach and Pastan 2020). However, the impact of gamma radiation on ADP-ribosyl transferase activity of PE24 moiety or its expression by expressing vector had been rarely assessed. Furthermore, the impact of divalent metal ions (e.g. Mg^2+^) present in phosphate-buffered saline used during extraction procedures and maintenance of in vitro cultivated cell cultures on PE toxin activity was not explored, knowing that, divalent cations (Mg^2+^ and Ca^2+^) could interact with PE toxin and inhibit its enzymatic activity (Blumentals et al. 1987; Özaslan et al. 2017).

In this study, we aimed to clone the nucleotide sequence that encodes catalytic domain Ib/III along with the furin cleavage site of exotoxin A retrieved from clinical *P. aeruginosa* isolates (PE24 moiety) in pET22b( +) for production inside *E. coli* BL21 (DE3). The ADP-ribosylation capability of the produced PE24 extract will be explored on NBAG and NAD^+^ prior to assessing its effect on in vitro cultured carcinoma cell lines (MCF-7, A375, Kasumi-1, and HEPG2) and assessing the impact of combining gamma radiation and paclitaxel with PE24 moiety on HEPG2 cells. The ADP-ribosylating capability of PE24 moiety on NBAG and NAD^+^ will also be examined after exposing *E. coli* BL21 (DE3) to different gamma radiation doses (5, 10, 15, and 24 Gy) to illustrate the impact of gamma radiation on the expression profile of *E. coli* BL21 (DE3) and activity of expressed PE24 moiety. Furthermore, the effect of divalent and monovalent metal ions on the ADP-ribosylating effect of PE24 moiety in the presence of NBAG and NAD^+^ will be assessed. Finally, we will explore the impact of conjugating radiotherapy at two gamma radiation doses 5 Gy and a single shot of 24 Gy and paclitaxel with the expressed PE24 moiety on the growth and proliferation of HEPG2 carcinoma cell lines.

## Material and methods

### *P. aeruginosa* isolates used in this study

The *P. aeruginosa* isolates, PA16 (Accession number: MZ713410.1) and PA22 (Accession number: MZ713405.1) were used in this study. Both isolates could express active *P. aeruginosa* exotoxin A capable of ADP-ribosylating NBAG (Morgan et al. 2021a, b). PA 16 and PA 22 were the sources of the exotoxin A gene (*tox*A) during DNA extraction and amplification of domain Ib/III of the *toxA* gene insert. Both PA16 and PA22 isolates were submitted to Culture Collection Ain Shams University (CCASU) which is a part of the World Data Centre for Microorganisms (WDCM) (https://ccinfo.wdcm.org/details?regnum=1186) (accessed on 23 October 2022) under the accession codes, CCASU-PA16, and CCASU-PA22, respectively.

### Primers used for PCR amplification and purification of PE24 toxin moiety

The PE24 moiety amplification primers included both restriction sites for *Nde*I and *Eco*R1 restriction enzymes in upstream and downstream strands, respectively. The designed primer sequences were Forward (5′-TTCGTCCGGCAGGGCATATGC-3′), and Reverse (5′-CCGGTCGCGGGAATTCCTTCA-3′). The genomic DNA purification kit (K0721, Massachusetts, USA) was used for DNA extraction and purification from PA16 and PA22 isolates (SI material and method 1). The purified DNA was used for amplification of PE24 moiety, where PCR reaction tubes contained eluted DNA (2 μl) mixed with previously mentioned primers (Willowfort, Birmingham, UK) at 5 μM (1 μl each), 12.5 μl of Dream*Taq* PCR master mix (2X) (Thermo Scientific, Massachusetts, USA) and deionized water (7 μl). The following program was used during the amplification procedure; 10 min of denaturation at 95 °C pursued by 30 cycles of 30 s denaturation at 95 °C, 30 s annealing at 59 °C, and 30 s extension at 72 °C. The program was terminated by a 7 min extension round at 72 °C. The amplified segment was visualized against a UV transilluminator using 1.2% agarose gel (molecular grade agarose, Bioline (Toronto, Canada), dissolved in 1X TBE buffer) and 100 bp DNA ladder termed Berus (Willowfort, Birmingham, UK) (Morgan et al. 2021a, b). Post amplicon UV visualization, Zymo Research DNA clean, and concentrate TM-25 (DCC-25) (California, USA) refined the amplified PE24 segments from reaction tube remnants to facilitate restriction by *Nde*I and *Eco*R1 enzymes (New England Biolabs, Massachusetts, USA) (SI material and method 2).

### pET22b( +) plasmid extraction from E. coli DH5α and purification for cloning procedure

The expression vector pET22b( +) was used for cloning and expression of PE24 moiety. The *E. coli* DH5α and expression strain *E. coli* BL21 (DE3) were obtained from (Novagen, Darmstadt, Germany). The *E. coli* DH5α harboring pET22b ( +) were cultivated on 100 μg/ml ampicillin-containing LB agar plates overnight at 37 °C. Using a sterile toothpick, 1 to 3 colonies were inoculated in sterile LB broth containing 100 μg/ml ampicillin. The broth was incubated on a shaking incubator (120 cycles per minute) at 37 °C for 3 h prior to plasmid extraction (Bangen et al. 2004). The Zymogen research Zyppy plasmid extraction Kit (D4036) (California, USA) was deployed for plasmid extraction (SI material and method 3). Using New England Biolabs loading dye, the extracted plasmid was visualized against a UV transilluminator using 1.2% agarose gel and Berus 1Kbp DNA ladder (Willowfort, Birmingham, UK). Excision of visualized plasmid band was performed using a sterile scalpel and the plasmid was purged from agarose gel using GeneJet gel extraction kit K0691 (Thermo Scientific, Massachusetts, USA) (SI material and method 4).

### Restriction of purified PE24 amplicon and plasmid by NdeI and EcoR1 restriction enzymes

Following purification procedures, PE24 amplicon-containing restriction sites of *Nde*I in its forward direction and *Eco*R1 in its reverse, and the pEt22b( +) was treated by restriction enzymes according to restriction digestion protocol provided by NEB (https://international.neb.com/protocols/2012/12/07/optimizing-restriction-endonuclease-reactions) (accessed on 23 October 2022). The restriction R_x_ tube contained 22 μl of purified amplicon or 15 μl of pET22b( +), mixed with 1 μl of *Nde*I (New England Biolabs, Massachusetts, USA), 5 μl of 10 × buffer, and 11 μl deionized water. The restriction R_x_ was incubated for 30 min at 37 °C; then, 1 μl of *Eco*R1 (New England Biolabs, Massachusetts, USA) was added. The tube was returned to the incubator at 37 °C for 2 h. To inactivate restriction enzymes, the restriction R_x_ tubes were heated for 90 s in a boiling water bath and then centrifuged for 15 s to get rid of the hue (Sambrook et al. 1989). A few microliters of the mixture were mixed with loading dye and visualized against a UV transilluminator using 1.2% agarose gel and Berus 1 Kb DNA ladder (Willowfort, Birmingham, UK). The remainder of the product was purified for ligation reaction using Zymo research DNA concentrate, and clean TM-25 (SI material and method 2).

### Ligation of restricted amplicon and pEt22b( +) to generate recombinant construct pET22b( +)-PA 16 and pET22b( +)-PA 22

The ligation reaction was initiated by incubating 12 μl of the refined restricted amplicon, 5 μl of purged restricted plasmid, 2 μl of T4 DNA ligase (Enzynomics, Daejeon, Republic of Korea), 2 μl of 10X buffer provided with ligase enzyme, and 15 μl deionized water overnight at 25 °C. This was performed according to NEB Protocol Ligation with *T4* DNA Ligase (https://international.neb.com/Protocols/0001/01/01/dna-ligation-with-t4-dna-ligase-m0202) (accessed on 23 October 2022). The ligated mixture was transformed into *E. coli* BL21 (DE3) (Sambrook et al. 1989).

### Ligated pET22b( +)-PA 16 and pET22b( +)-PA 22 construct CaCl_2_ and heat shock transformation into E. coli BL21(DE3)

The ligated constructs pET22b( +)-PA 16 and pET22b( +)-PA 22 were introduced into *E. coli* BL21 (DE3) by CaCl_2_ and heat shock transformation technique. First, competent *E. coli* BL21 (De3) cells were prepared where 600 μl of an overnight *E. coli* BL21 (DE3) culture in LB broth was inoculated in supper optimal broth (SOB) and re-incubated at 37 °C in a shaking incubator (250 rpm) until OD 0.6 (around 4 h) (Sambrook et al. 2006). The broth was 6000 × g centrifuged for 10 min at 4 °C to sediment bacterial cells. After broth decantation, the bacterial cake was loosened with 5-ml ice-cold transformation buffer containing 20% glycerol and maintained in an ice bucket for 30 min prior to transformation. The bacterial/transformation buffer suspension was then divided into 200 μl aliquots in a sterile 2 ml Eppendorf tube; each of these aliquots was supplemented with 20 μl of the ligated construct. The bacterial aliquots Eppendorfs were maintained in an ice bucket for 30 min before placing them in a 42 °C water bath for 90 s. Afterward, the Eppendorfs were rapidly cooled down by maintaining them in an ice bucket for 2 min. The bacterial/transformation buffer suspensions were replenished by 800 μl of super optimal broth with catabolite repression broth (SOC) and kept in a shaking incubator (250 rpm) at 37 °C overnight. The broths were spread on 100 μg/ml ampicillin-containing LB agar plates to allow growth and selection of the growth of transformant cells only (Sambrook et al. 2006; Chang et al. 2017).

### Colony PCR, restriction enzymes digestion for pET22b( +)-PA 16/PA 22 constructs and sequencing after introduction into E. coli BL21 (DE3)

To confirm insert introduction inside constructs and their uptake by *E. coli* BL21 (DE3), colony PCR was implemented. A total of 2–3 colonies from bacterial growth visible on LB plates were suspended in sterile deionized water and mixed with previously mentioned primers and *Taq* master mix (2X, Thermo Scientific, Massachusetts, USA) in sterile PCR Eppendorf. The tube was then inserted in a thermocycler (CG Palm-Cycler™, genetix biotech, New Delhi, India), and the PCR reaction program used to amplify PE24 moiety was applied. Following the amplification, the PCR reaction product was visualized by a UV transilluminator using 1.2% agarose gel and Berus 100 bp DNA ladder (Morgan et al. 2021a, b). A PCR reaction product was sent out for sequencing analysis at Colors lab medical laboratories, Clinilab Co. Cairo, Egypt. The first-generation Sanger sequencing was performed, and the sequence obtained was blasted against the National Center of Biotechnology Information (NCBI) GenBank protein and nucleotide sequences using MegaX software version 10.0.5. The plasmid was also extracted from transformed *E. coli* BL21 (DE3) and restricted by *Nde*I and *Eco*R1 enzymes and the insert was visualized against UV transilluminator using 1.2% agarose gel and Berus 1 Kb DNA ladder (Sambrook, J. 2001; Morgan et al. 2021a, b).

### Protein expression in E. coli BL21 (DE3) and SDS-PAGE to confirm protein expression

Both pET22b( +)-PA 16 and pET22b( +)-PA 22 were introduced into *E. coli* BL21 (DE3), cultivated in 0.5 mM IPTG and 100 μg ampicillin-containing LB broth, and maintained inside a shaking incubator overnight at 37 °C. The resultant bacterial broths were centrifuged at 7000 × g for 20 min. After broth removal, the bacterial cake was dissolved in 10 mM Tris HCl, 300 mM NaCl, 50 mM NaH_2_PO_4_, 1 mg/ml lysozyme (Thermo Scientific, Massachusetts, USA), and 10 mM imidazole-containing lysis buffer and sonicated in cooling ice for 10 min. The bacterial/lysis buffer suspension was 7000 × g centrifuged for 20 min to sediment cell debris and the supernatant was mixed with 1 × SDS lysis buffer (1%SDS, β-mercaptoethanol, glycerol, Tris HCl) and boiled for 5 min prior to loading into 15% acrylamide gel to perform protein electrophoresis using sodium dodecyl sulfate-poly acryl amide gel and Laemmli buffer system (Costas 1995). The gel analysis was performed using SynGene Gene Tools-file version 4.3.10.0-serial no. 9999*9999*license*mpcsdb23q in faculty of science, Ain Shams University, Cairo, Egypt. To test the ADP-ribosyl transferase activity of the expressed protein, the supernatant obtained after the removal of the cell debris was dialyzed twice against 0.01 M Tris buffer using a cellulose dialysis bag of 12–13 KDa cutoff (Frey Scientific, Inc. USA). The dialyzed protein extract concentration was adjusted against bovine serum albumin (BSA) where the absorbance of the serial dilution of BSA mixture at 280 nm was plotted against concentrations (20, 50, 100, 250, 500, 1000, 2000, and 3000 µg/ml) and absorptivity was computed. The concentration of the dialyzed protein extract was computed using this formula;

$$\mathrm{Protein}\;\mathrm{concentration}(\frac{\mathrm{mg}}{\mathrm{ml}})=\frac{\mathrm{Sample}\;\mathrm{Absorbance}\;\mathrm{at}\;280\mathrm{nm}}{\mathrm{absorbitivity}\times\mathrm b}$$

With *b* being the quartz cuvette path length in cm (Simonian et al. 2006; Burgess 2009).

### NBAG ADP-ribosylation by PE24 extracts using different spectroscopic techniques (UV, FTIR, and C.^13^-NMR)

The capability of PE24 extracts expressed by pET22b( +)-PA16 and pET22b( +)-PA22 harboring *E. coli* BL21 (DE3) to ADP ribosylate nitrobenzylidine aminoguanidine (NBAG) were elucidated by UV spectroscopy (Morgan et al. 2021a, b). The Jasco V-670 double-beamed UV/VIS spectrophotometer was utilized to record fluctuation, shifts, and absorbance maxima in the absorbance spectrums of reaction tubes among UV wavelength range (200–500 nm). A total of 150 μl from PE24 moieties protein extracts of 300 μg/ml concentration was incubated with dithioerithritol (DTE) of 0.01 M (200 μl), tris acetate of 2 mM (pH 8, 200 μl), and sterile distilled water (400 μl) at 37 °C for 30 min. Afterward, the tube was supplemented with nicotinamide adenine dinucleotide (NAD^+^) of 4 mM (400 μl), 1200 μl of previously synthesized and lyophilized 10 mM NBAG (Morgan et al. 2021a, b) dissolved in 0.1 N HCl, and sterile distilled water (400 μl). The tube was maintained in room temperature (25 °C) for 1 h prior to absorbance spectrum observation. A reference tube was used during observation of the absorbance spectrum containing all the reaction tube ingredients except PE24 moieties extract. The absorbance maxima computed were used to elucidate ADP-ribosylated NBAG formed by PE24 moieties (Soman et al. 1983; Morgan et al. 2021a, b). Soman et al. demonstrated that change in absorbance by 0.1 unit is equivalent to formation of 5 × 10^–2^ μmol/ml ADP-ribosylated NBAG wherefore:


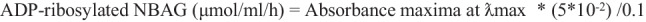

$$\mathrm{Percent}\;\mathrm{ADP}-\mathrm{ribosylated}\;\mathrm{NBAG}\;\mathrm{formed}=\frac{\left(\mathrm{conc}.\;\mathrm{of}\;\mathrm{ADP}\;\mathrm{ribosylated}\;\mathrm{NBAG}\;\mathrm{formed}\right)}{\left(\mathrm{original}\;\mathrm{conc}.\;\mathrm{of}\;\mathrm{NBAG}\right)}\times100$$

$$\mathrm{Enzymatic}\;\mathrm{activity}\;\mathrm{of}\;\mathrm{PE}24\;\mathrm{moiety}\;\mathrm{per}\;\mathrm{hour}\left(\frac{\mathrm U}{\mathrm h}\right)=\mathrm{ADP}-\mathrm{ribosylated}\;\mathrm{NBAG}\;\left(\mathrm\mu\mathrm m\mathrm o\mathrm l/\mathrm{ml}/\mathrm h\right).$$

The reaction mixture was also investigated by FTIR spectroscopy using Bruker FITR spectrometer (Vertex 70 v vacuum) across the wavenumber range 400–4000 cm^−1^. The reaction mixture was spotted on a clean glass slide and air-dried at room temperature overnight. The reaction spot was placed in Bruker Vertex 70 v vacuum FTIR spectrometer and the spectrum generated was analyzed. Furthermore, C^13^-NMR was also used to elucidate the structural changes in NBAG structure after treatment by PE24 moiety. The remnant of the reaction spot was scratched gently from the glass slide using a sterile stainless blade and the powder was dissolved in dimethylsulfoxide-d6 (DMSO-D6) and examined overnight to obtain the spectrum. Bruker NMR located at the central labs of the Faculty of Pharmacy, Ain Shams University (Cairo, Egypt), was used to conduct this assay (Morgan et al. 2021a, b).

### High performance liquid chromatography (HPLC) to determine PE24 moiety ADP-ribosylating action

ADP-ribosyl transferase action of PE24 moieties expressed by pET22b( +)-PA 16 and pET22b( +)-PA 22 harboring *E. coli* BL21 (DE3) was elucidated by HPLC as described by Morgan et al. (2021a, b). To initiate the ADP-ribosyl transferase reaction, Tris–acetate (pH 8, 0.2 M), NAD.^+^ (4 mM), DTE (0.02 M), and NBAG (10 mM) were mixed with 300 µg/ml of PE24 moieties extract. Ten percent trichloroacetic acid (TCA) was introduced into the reaction tube following 3 h incubation at room temperature (25 °C). A reference tube containing all reaction ingredients except for PE24 moieties extract was used as a standard tube to generate a standard HPLC chromatogram plot. Both reaction tubes and reference tubes were injected into Waters 2690 Alliance HPLC system equipped with Waters 996 photodiode array detector, located in Nawah Scientific Inc. laboratories, Cairo, Egypt. The column used for the analysis was C18 Inertsil of 4.6 × 250 mm and 5 μm particle size. Prior to injection, the samples were passed through a filter syringe of 0.22 μm size. A total of 100 μl of the reaction mixture was injected into the C18 column with a 1 ml/min flow rate. The mode of elution was gradient with alternating concentrations of water and methanol as mobile phase (Table S1) at ambient temperature. The chromatograms were recorded at a single wavelength of 301 nm, and the peak area was determined. The ADP ribosylated NBAG produced by PE24 moieties were calculated relative to the original amount of NBAG (Soman et al. 1983; Kupiec 2004)

$$\mathrm{Conc}.\;\mathrm{of}\;\mathrm{unknown}=\frac{\mathrm{Area}\;\mathrm{of}\;\mathrm{unknown}}{\mathrm{Area}\;\mathrm{of}\;\mathrm{known}}\times\mathrm{conc}.\;\mathrm{of}\;\mathrm{known}$$

$$\mathrm{ADP}-\mathrm{ribosylated}\;\mathrm{NBAG}\;\mathrm{formed}\;\mathrm{per}\;\mathrm{hour}\;\left(\mathrm\mu\mathrm m\mathrm o\mathrm l/\mathrm{ml}/\mathrm h\right)=\mathrm{total}\;\mathrm{ADP}-\mathrm{ribosylated}\;\mathrm{NBAG}\;\mathrm{formed}\;\mathrm{in}\;3\;\mathrm h\left(\mathrm\mu\mathrm m\mathrm o\mathrm l/\mathrm{ml}\right)/3$$

$$\mathrm{Enzymatic}\;\mathrm{activity}\;\mathrm{of}\;\mathrm{PE}24\;\mathrm{moiety}\;\mathrm{per}\;\mathrm{hour}\left(\frac{\mathrm U}{\mathrm h}\right)=\mathrm{ADP}-\mathrm{ribosylated}\;\mathrm{NBAG}\;\left(\mathrm\mu\mathrm m\mathrm o\mathrm l/\mathrm{ml}/\mathrm h\right)$$

### Influence of gamma irradiation on expression of recombinant PE24 moiety and its ADP-ribosylating activity

The effect of irradiating pET22b( +)-PA 16 harboring *E. coli* BL21 (DE3) by low-dose gamma rays (5,10,15, 24 Gy) on the expression and production of PE24 moieties was explored. Fresh LB bacterial broths at exponential phase supplemented with 100 μg/ml ampicillin were irradiated at 5, 10, 15, and 24 Gy emitted from Cs^137^ source of Canadian Gamma Cell-40 located inside the Egyptian Atomic Energy Authority, National Centre for Radiation Research and Technology (NCRRT), Cairo, Egypt. The irradiation dose rate applied was 0.633 rad/s (Morgan et al. 2021a, b). Following bacterial broth irradiation, 0.5 mM IPTG was added and bacterial broths were incubated in a shaking incubator at 250 rpm at 37 °C overnight prior to protein extraction as described above. SDS page and ADP-ribosylation capabilities were examined as previously discussed to demonstrate the influence of low-dose gamma rays irradiation on PE24 expression of pET22b( +)-PA 16 by *E. coli* BL21 (DE3).

### Impact of metal salts on ADP-ribosyl transferase activity of PE24 moiety by UV spectroscopy and HPLC

To explore the impact of metal salts on the ADP-ribosylating activity of PE24 moiety, reaction tubes containing NBAG, NAD^+^, and Tris–acetate were supplemented with the 10 mM of sodium chloride, zinc chloride, magnesium chloride, and Magnesium sulfate prior to the addition of PE24 moiety mixture and incubation. Following the addition of PE24 moiety extract (pET22b( +)-PA16 harboring *E. coli* BL21 (DE3)) to reaction tubes, the reaction was analyzed by UV spectroscopy and HPLC as previously described (Morgan et al. 2021a, b).

### Cytotoxicity of PE24 moiety on HEPG2 and MCF-7 cell lines alone and in combination with paclitaxel by MTT cell proliferation and cytotoxicity test

ATCC-grade human hepatocellular carcinoma (HEPG2) and human breast carcinoma (MCF-7) cell lines were obtained from Nawah Scientific Inc. laboratories, Cairo, Egypt. The cells were cultivated in RPMI 1640 media (Lonza Bioscience, Basel, Switzerland) fortified with 10% complement inactivated fetal bovine serum (FBS) (Gibco Sera, USA), 100 mg/ml streptomycin, 100 units/mL of penicillin, and 80 mg/ml gentamicin and maintained in 37 °C humidified 5% CO_2_ incubator. At optimal confluence (95% confluence, 48 h post-incubation), the old media was discarded, cells monolayers were washed twice using fertile sterilized phosphate-buffered saline (PBS) (Lonza Bioscience, Basel, Switzerland), and 2000 μl of Trypsin–EDTA was added. The flasks were returned to the CO_2_ incubator at 37 °C for 5 min, then, vigorously shaken facilitating cell detachment. The detached cells were supplemented with fresh RPMI media containing 10% FBS and antibiotics mixture and pipetted into sterile 96 cell culture plates wells prior to transfer into 96 well-plate (Cell star, Scientific, Inc. USA), 100 μl (5 × 10^3^ cells) per well. The plates were re-incubated in the previously mentioned conditions for 24 h until confluent cell monolayers were observed in wells (Morgan et al. 2019; Morgan et al. 2021a, b). Culture media in plates were rinsed off, plates were washed by PBS and fresh RPMI 1640 media with 10% FBS were pipetted in each well along with 50 μl of PE24 moieties extract (pET22b( +)-PA16 harboring *E. coli* BL21 (DE3)) and paclitaxel (Fresenius Kabi Oncology, Thailand) (positive control) at concentrations (3, 10, 30, 100, 300 μg/ml). The Tris buffer was pipetted into some wells as a negative control. Both HEPG2 and MCF-7 cells were also treated with PE24 moieties extract at concentrations (3, 10, 30, 100, 300 μg/ml) in combination with low-dose paclitaxel 5 μg/ml.

Following the treatment of the cells, the plates were re-incubated for 48 h prior to the application of 3-(4,5-dimethylthiazol-2-yl)-2,5-diphenyltetrazolium bromide (MTT) cytotoxicity and proliferation assay. First, 50 mg of MTT powder (ACTOS) was dissolved in 10 ml PBS. The pre-existing media in plate wells were rinsed off, plate irrigation by PBS (2 times) was performed to get rid of any media remnants, and 15 μl of freshly formulated MTT solution was pipetted into each well. The plates were kept inside the incubator at 37 °C for 3 h. Afterward, formazan crystals formed in plate wells were solubilized by 100 μl dimethylsulfoxide (DMSO) to allow absorbance measurement at 600 nm using Humareader HS microtiter plate reader (Morgan et al. 2021a, b). The percent cytotoxicity, cell viability, and IC50 were calculated as follows:

$${HEPG2\;or\;MCF7\;\boldsymbol c\boldsymbol e\boldsymbol l\boldsymbol l\boldsymbol{\mathit\;}\boldsymbol v\boldsymbol i\boldsymbol a\boldsymbol b\boldsymbol i\boldsymbol l\boldsymbol i\boldsymbol t\boldsymbol y\;}\%=\frac{\mathrm{PE}24\;\mathrm{Treated}\;(\mathrm{HEPG}2\;Or\;\mathrm{MCF}7)\;\mathrm{cell}\;\mathrm{absorbance}}{\mathrm{untreated}\;(\mathrm{HEPG}2\;\mathrm{or}\;\mathrm{MCF}7)\;\mathrm{cell}\;\mathrm{absorbance}\;(\mathrm{control})}\times100$$

$$HEPG2\;or\;MCF7\;\boldsymbol c\boldsymbol e\boldsymbol l\boldsymbol l\boldsymbol{\mathit\;}\boldsymbol c\boldsymbol y\boldsymbol t\boldsymbol o\boldsymbol t\boldsymbol o\boldsymbol x\boldsymbol i\boldsymbol c\boldsymbol i\boldsymbol t\boldsymbol y\;\%=100-cell\mathit\;viability\%$$

### Cytotoxicity of PE24 moiety on A375: human melanoma cells by sulforhodamine B assay (SRB)

ATCC grade A375: human melanoma cells retrieved from Nawah Scientific Inc., Cairo, Egypt, in Dulbecco's modified eagle medium (DMEM) (Gibco Thermo Fisher, Massachusetts, USA) fortified by 100 mg/ml streptomycin, 100 units/ml of penicillin and 10% FBS were used to conduct SRB cytotoxicity assay. A375 cells suspended in 10% FBS-containing DMEM were pipetted into 96 well cell culture plates, 100 μl in each well, and the inoculated plates were maintained inside 37 °C humidified incubators with 5% CO_2_ till optimal confluence. A total of 50 μl from PE24 moiety extract (pET22b( +)-PA16 harboring *E. coli* BL21 (DE3)) and doxorubicin (positive control) were pipetted into plate wells at concentrations (0.01, 0.1, 1, 10, 100 μg/ml) and returned to the CO_2_ incubator for 72 h post 72 h. During the incubation period, the media was rinsed off, plates were irrigated with PBS twice and 10% trichloroacetic acid (100 μl) was used for cell fixation at 4 °C (~ 1 h). Afterward, trichloroacetic acid was rinsed off and plates were irrigated with sterile distilled water 5 times prior to the addition of 70 μl SRB solution (0.4% W/V). The plates were kept in the dark at 25 °C for 10 min before removal of SRB solution by 1% acetic acid (3 times) and left to air-dry overnight. To solubilize protein-bound SRB, 10 mM tris (150 μl) is added into each well and the absorbance of the produced solution was scored at 540 nm by BMG LABTECH®-FLUOstar Omega microplate reader (Ortenberg, Germany) in Nawah Scientific Inc. (Cairo, Egypt) (Morgan et al. 2021a, b). The A375 cell viability% was computed:

$$A375\;\boldsymbol c\boldsymbol e\boldsymbol l\boldsymbol l\boldsymbol\;\boldsymbol v\boldsymbol i\boldsymbol a\boldsymbol b\boldsymbol i\boldsymbol l\boldsymbol i\boldsymbol t\boldsymbol y\;\%=\frac{\mathrm{PE}24\;\mathrm{Treated}\;\;\mathrm A375\;\mathrm{cells}\;\mathrm{absorbance}}{\mathrm{untreated}\;\mathrm A375\;\mathrm{cells}\;\mathrm{absorbance}\;(\mathrm{Control})}\times\;100$$

### Cytotoxicity of PE24 moiety on OEC: normal oral epithelial cells and Kasumi-1: acute monocytic leukemia (AML) cells using WST-1 cytotoxicity assay

ATCC grade OEC: normal oral epithelial cells grown in DMEM media (Lonza, Basel, Switzerland) with 10% FBS, 100 units/ml of penicillin and 100 mg/ml streptomycin and Kasumi-1: acute monocytic leukemia (AMl) cells cultivated in RPMI media (Lonza, Basel, Switzerland) with 10% FBS and same antibiotic solution were obtained from Nawah Scientific Inc. Cairo, Egypt. Both cells were used to assess PE24 moiety (pET22b( +)-PA16 harboring *E. coli* BL21 (DE3)) cytotoxicity using WST-1 cytotoxicity assay. Abcam WST-1 assay kit (ab155902 WST-1 cell proliferation reagent) was utilized during the conduction of the cytotoxicity assay. Fresh media containing different concentrations of PE24 moiety extract and doxorubicin (0.01, 0.1, 1, 10, and 100 μg/ml) were pipetted (50 μl) into cell culture plates exhibiting confluent growth. After treatment, the plates were maintained inside humidified 5% CO_2_ incubator at 37 °C for 48 h. The plates media were rinsed off and 10 μl of WST-1 reagent was pipetted to measure the absorbance of suspension at 450 nm using a BMG LABTECH-FLUO star Omega microtiter plate reader (Ortenberg, Germany) located in Nawah Scientific Inc., Cairo, Egypt (Alaufi et al. 2017). The cell viability percentage was computed as follows:

$$OEC\;or\;Kasumi1\;\boldsymbol c\boldsymbol e\boldsymbol l\boldsymbol l\boldsymbol\;\boldsymbol v\boldsymbol i\boldsymbol a\boldsymbol b\boldsymbol i\boldsymbol l\boldsymbol i\boldsymbol t\boldsymbol y\;\%=\frac{\mathrm{PE}24\;\mathrm{Treated}\;(\mathrm{OEC}\;\mathrm{or}\;\mathrm{Kasumi}1)\;\mathrm{cell}s\;\mathrm{absorbance}}{\mathrm{untreated}\;(\mathrm{OEC}\;\mathrm{or}\;\mathrm{Kasumi}1)\;\mathrm{cell}s\;\mathrm{absorbance}\;(\mathrm{control})}\times100$$

### Cytotoxicity of PE24 moiety on HEPG2 cells pre-treated with low-dose gamma radiation by MTT assay

ATCC grade HEPG2 cells maintained in RPMI media with 10% FBS were used to test the impact of irradiating cancer cell lines with low doses of gamma radiation prior to treatment with PE24 moiety and assessing overall cytotoxicity. The cells were maintained and culture plates were cultivated as described above. When the plates reached optimum confluence, the plates were irradiated separately at two different doses, 5 Gy and one shot 24 Gy doses emitted by Cs^137^ source of Canadian Gamma Cell-40 located inside Egyptian Atomic Energy Authority, National Centre for Radiation Research and Technology (NCRRT), Cairo, Egypt. Following plates irradiation, old media were rinsed off, plates were irrigated with PBS, and fresh RPMI 1640 media with 10% FBS were pipetted into each well. The plates were re-incubated in humidified 5% CO_2_ incubator at 37 °C. After 24 h period of incubation, the media were rinsed off, and wells were irrigated with PBS and new media along with PE24 moiety extract (pET22b( +)-PA16 harboring *E. coli* BL21 (DE3)) at concentrations 3, 10, 30, 100, and 300 μg/ml were pipetted into wells of both plates. The plates were maintained inside humidified 5% CO_2_ incubator at 37 °C for 48 h. Afterward, the cytotoxicity was assessed by MTT assay as described above (Morgan et al. 2021a, b).

### Cytotoxicity of PE24 moiety on HEPG2 cells in combination with low-dose gamma radiation by SRB assay

The ATCC-grade HEPG2 cells maintained in DMEM media with 10% FBS were used to test the overall cytotoxic effect of PE24 in combination with 2 gamma radiation doses, low-dose 5 Gy and one shot 24 Gy on cultivated cells. The cells were cultured in regular cell culture plates and then exposed to PE24 moiety extract (pET22b( +)-PA16 harboring *E. coli* BL21 (DE3)) at (0.01, 0.1, 1, 10, and 100 μg/ml). The plates were irradiated separately at 5 Gy and one shot 24 Gy after the cell’s treatment and an SRB assay was conducted as described above (Morgan et al. 2021a, b).

### NCBI accession code

The sequence of cloned PE 24 moiety is deposited on the NCBI GenBank database under the accession code, OP889245.

### Statistical analysis

The regression analysis and *R*^2^ value for the calculation of the IC 50 values were performed on SigmaPlot Version 10.0 Built 10.0.0.54. The *P* values for cell viability percentage post-exposure to PE24 moiety extract were calculated using positive (paclitaxel and doxorubicin) and negative (Tris buffer) control values using ANOVA: Single Factor data analysis assay in excel.

## Results

### PCR amplification of PE24 toxin moiety form P. aeruginosa isolates and pET22b( +) plasmid extraction from E. coli DH5α

Figure S1 elucidates the electrophoresed PE24 moiety amplicons (~ 600–700 bp) amplified from several *P. aeruginosa* isolates (PA 16, PA 22, PA 39, PA 35, and PA 19) and pET22b ( +) plasmid extracted from *E. coli* DH5α. *E. coli* BL21 (DE3) chromosomal DNA was also extracted and screened along with *P. aeruginosa* isolates for PE24 gene segment presence to confirm the purity of the isolate prior to cloning. The 5000 bp band of pET22b( +) was purified along with the products as shown in Fig. S2.

### Colony PCR and appearance of insert post ligation of the restricted pET22b( +) and PE24 product using T4 DNA ligase

The purified pET22b( +) and PE24 were restricted as shown in Fig. S3. Introduction of pET22b( +)-PA 16/PA 22 construct into *E. coli* BL21 (DE3) was successful as shown by visible colonies in 100 μg/ml ampicillin-containing LB agar plates (Fig. S4). Figure S5 illustrates the electrophoresed colony PCR products of pET22b( +)-PA 16 and pET22b( +)-PA 22 transformed *E. coli* BL21 showing amplified PE24 insert at ~ 600–700 bp. Furthermore, PE24 insert (~ 600–700 bp) were electrophoresed after *Nde*I and *Eco*R1 digestion of pET22b( +)-PA 16 construct isolated from transformed *E. coli* BL21 (DE3) (Fig. S6).

### Sequencing analysis of colony PCR product for pET22b( +)-PA 16 insert

The blasted nucleotide segment retrieved from pET22b( +)-PA 16 (NCBI accession, OP889245) was identical by 98.74%, 98.11%, and 98.11% to *P. aeruginosa* exotoxin A protein sequences of the following accessions, MCO3035995.1, KPE31189.1, HBO9324115.1, respectively. It was also identical by 98.74%, 98.11%, 98.09%, and 98.74% to exotoxin A protein sequences of these accessions, VTQ12157.1, MCO2331336.1, NQD10526.1, and WP_033888293.1, respectively. Moreover, the nucleotide sequence retrieved from pET22b( +)-PA 16 construct was 99.58% identical to *P. aeruginosa* exotoxin A nucleotide sequence retrieved from the following accessions, MH373640.1, MZ408288.1, MW732028.1, and MW717584.1. The bootstrap trees generated for translated protein and nucleotide sequence alignments are presented in Figs. S7 and S8, respectively.

### SDS PAGE for expressed PE24 moiety post induction of pET22b( +)-PA 16 and pET22b( +)-PA 22 transformed E. coli BL21 (DE3) with IPTG

Figures S9–S13 show total proteins retrieved from *E. coli* BL21 (DE3) cells when transformed with both empty and cloned pET22b( +) with or without 0.5 mM IPTG induction. It was noted that a protein band around molecular weight 30 KDa (29 to 30 KDa) appeared only in induced *E. coli* BL21 (DE3) harboring pET22b( +)-PA 16 and pET22b( +)-PA 22 constructs and absent in non-induced *E. coli* BL21 (DE3) that are transformed by empty pET22b( +). This suggested the expression of PE24 by transformed *E. coli* BL21 (DE3) under IPTG stimulation. The raw volume and raw volume % of the protein band at 0.5 mM IPTG induction is recorded in Table S2.

Influence of irradiation by low-dose gamma rays on PE24 moiety expression by transformed *E. coli* BL21 (DE3).

The protein of molecular weight ~ 30 KDa was absent among protein extracts of *E. coli* transformed with pET22b( +)-PA 16 irradiated by 24 Gy as shown in Figs. S9 and S13. It was also noted that raw volume% was reduced after low-dose gamma irradiation of pET22b( +)-PA 16 transformed *E. coli* BL21 (DE3) (Table S2).

### PE24 moiety ADP-ribosylating activity explored by different spectroscopic techniques (UV, FTIR, and C.^13^-NMR)

A notable displacement in NABG *λ*_max_ was observed from 317 nm to approximately 365 nm (Fig. S14). The changes in concentration of NBAG post-exposure to PE24 moiety were recorded in Table S3. The enzymatic activity of PE24 moiety per hour (U/h) from *E. coli* BL21 (DE3) extracts is equivalent to conc. of ADP ribosylated NBAG formed per hour (μmol/ml/h). The highest enzymatic activity of PE24 moiety (U/h) retrieved from extracts of pET22b( +)-PA 16 harboring *E. coli* BL21 (DE3) was 0.482 whereas enzymatic activity of PE24 moiety (U/h) retrieved from extracts of pET22b( +)-PA22 harboring *E. coli* BL21 (DE3) was 0.542.

**Fig. 1:**
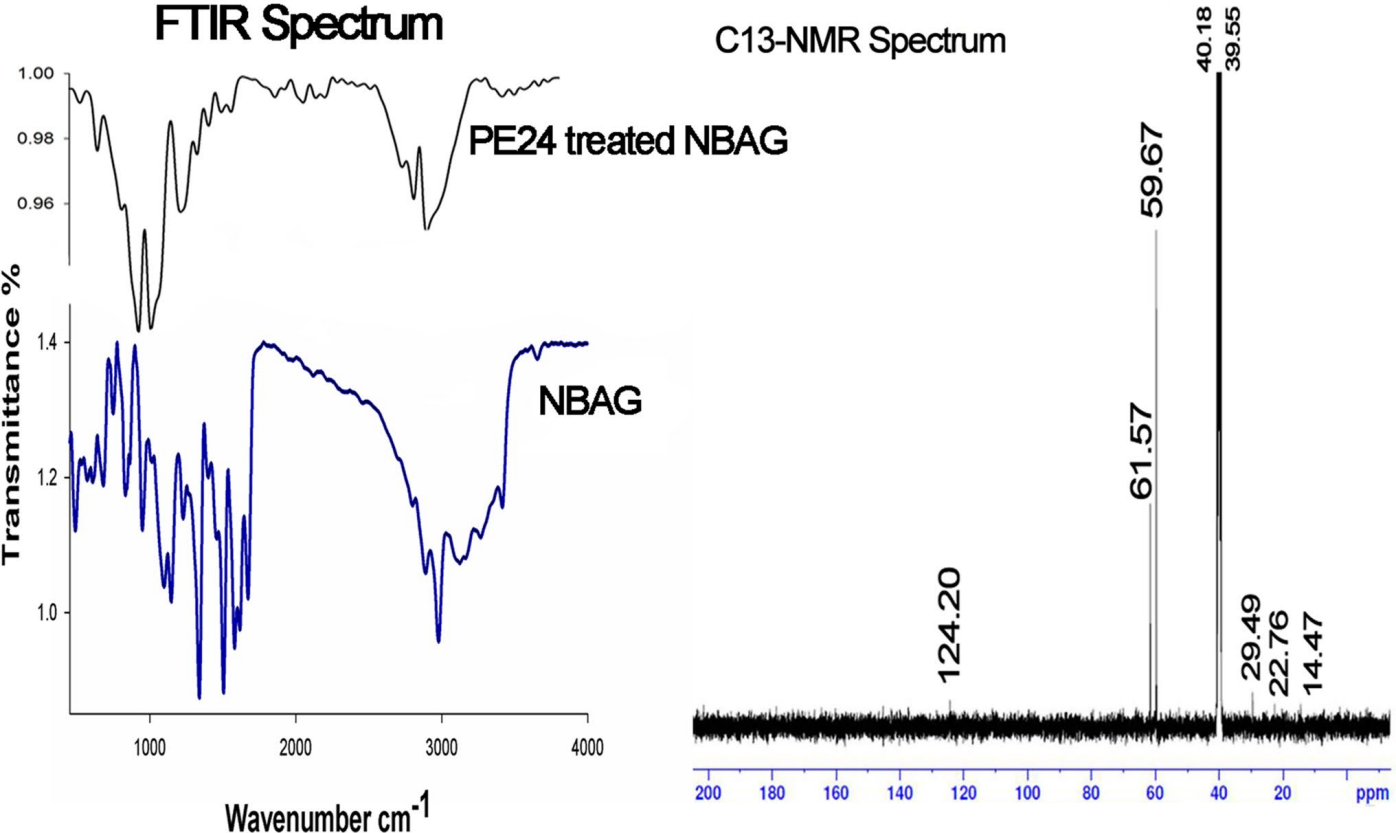
FTIR and ^13^C-NMR spectrum for NBAG reaction tube post exposure to PE24 extract

FTIR spectrum of spotted reaction revealed prominent structural changes from the original NBAG structure. The stretching band of NBAG located at 3500 cm^−1^ has been shifted to a lower wavenumber and its broadness was reduced to 2936–3020 cm^−1^, a forked peak located at approximately 900–1000 cm^−1^ and small peaks at 2314–2109 cm^−1^ appeared (Fig. 1). The peaks located 1300–1500 cm^−1^ remained in both spectrum before and after treatment however their transmittance was reduced (Fig. 1). These structural changes suggested ADP ribosylation of NBAG. Additionally, C^13^-NMR denoted the presence of a strong band at 124, 61.5, 59, 40.18, 29.49, and 22.76 ppm which differed from the original structure of NBAG (Fig. 1).

### PE24 moiety ADP-ribosylation of NBAG elucidated by HPLC

**Fig. 2:**
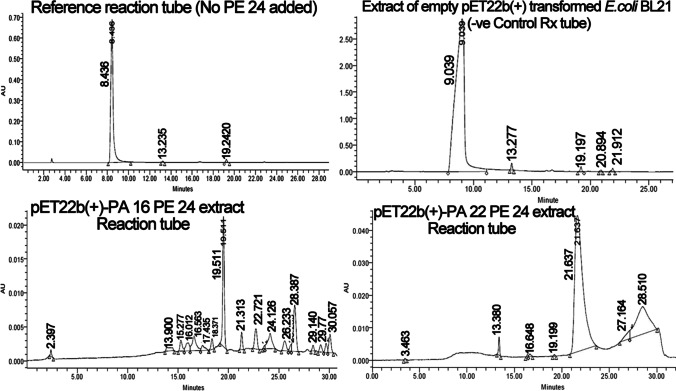
HPLC chromatogram for NBAG reaction tubes treated by PE24 extract

Table 1 computes ADP-ribosylated NBAG concentration formed in reaction tubes supplemented with PE24 moieties. Figure 2 sights the prominent changes in the reaction tube post-exposure to PE24 moieties extract. In the reference tube, the NBAG compound appeared at retention time (~ 9 min.). In reaction tubes prominent shift to higher retention time and emergence of new peaks around retention time (21–27 min) was observed suggesting ADP-ribosylation of NBAG. PE24 moiety retrieved from pET22b( +)-PA 16 harboring *E. coli* BL21 (DE3) scored an enzymatic activity of 0.6 U/h while pET22b( +)-PA 22 harboring *E. coli* BL21 (DE3) PE24 moiety exerted an enzymatic activity of 1.08 U/h. In terms of total percentage of ADP-ribosylated NBAG formed post exposure to PE24 moiety, pET22b/PA 16 PE24 moiety extract ADP-ribosylated NBAG by 16% whereas pET22b( +)/PA 22 PE24 toxin extract ADP-ribosylated NBAG by 34%.

**Table 1:** ADP ribosylated products of NBAG formed post exposure to pET22b( +)-PA16/PA22 PE24 moiety extract by HPLC

Transformed *E. coli* Bl22	Retention time at 301 nm	Peak Area	ADP-ribosylated NBAG in (M) in 3 h incubation period	ADP-ribosylated NBAG in μmol/ml in 3 h incubation period	ADP-ribosylated NBAG (μmol/ml/h)	Total Percent of ADP ribosylated NBAG in 3 h. (M)	Enzymatic activity of PE24 moiety per h. (U/h)
pET22b( +)-PA16 NBAG peak	19.511	2,124,580	0.01				
pET22b-PA16 New peaks	21.313	27,270	1.2 × 10^–4^				
	22.721	51,072	2.4 × 10^–4^				
	23.6	4210	1.9 × 10^–5^				
	24.126	65,606	3 × 10^–4^				
	25.577	30,045	1.4 × 10^–4^				
	26.233	10,613	4.9 × 10^–5^				
	26.567	128,424	6 × 10^–4^				
	28.387	9173	4.3 × 10^–5^				
	29.14	16,806	7.9 × 10^–5^				
	29.677	18,450	8.6 × 10^–5^				
	30.057	40,498	1.9 × 10^–4^				
	Total		0.002	2	0.667	18.9%	0.6
pET22b( +)-PA22 NBAG peak	21.637	2,505,171	0.01				
pET22b-PA22 New peaks	27.164	81,217	3.2 × 10^–4^				
	28.51	787,280	3.1 × 10^–3^				
	Total		0.0034	3.4	1.13	34.66%	1.08

ADP ribosylated product formed: area of unknown new peak/area of NBAG peak × conc. of NBAG in M; percent of ADP ribosylated products formed: total conc. of ADP ribosylated product formed/conc. of NBAG × 100: ADP-ribosylated NBAG formed per hour (μmol/ml/h): ADP-ribosylated NBAG in μmol/ml in 3 h/3: enzymatic activity of PE24 moiety per hour (U/h) = ADP-ribosylated NBAG formed per hour (μmol/ml/h)

### PE24 moiety ADP-ribosylating activity after irradiating pET22b( +)-PA 16 harboring E. coli BL21 and the addition of different metal salts determined by UV spectroscopy and HPLC

**Fig. 3:**
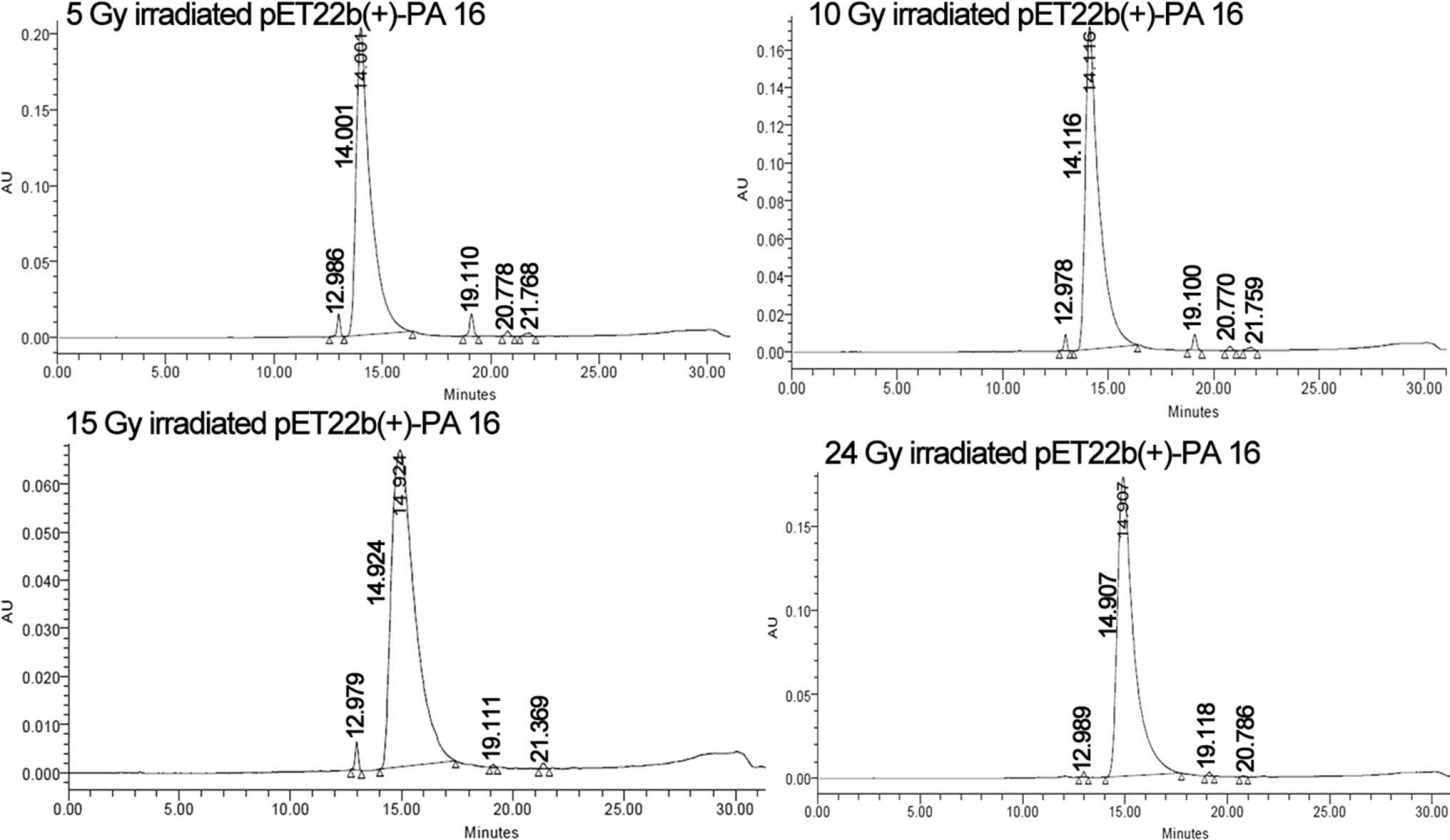
HPLC chromatogram for NBAG reaction tubes treated by PE24 extract retrieved from gamma irradiated pET22b( +)-PA16 transformed *E. coli* BL21 (DE3)

The PE24 extract from gamma-irradiated pET22b( +)-PA 16 harboring *E. coli* BL21 (DE3) failed to produce noticeable amounts of ADP-ribosylated product in a dose-dependent manner (Tables S4 and S5). Irradiating *E. coli* broth prior to induction of expression and extraction of PE24 moieties at 5, 10, 15, and 24 Gy was associated with an eminent reduction in PE24 moiety enzymatic activity. Exposure to 5, 10, 15 and 24 Gy was associated with PE24 moiety enzymatic activity of 0.09, 0.067, 0.012, and 0.0087, U/h respectively as recorded by HPLC chromatogram. This was reflected by an eminent reduction in the amount of ADP-ribosylated NBAG by 85%, 89%, 97.9%, and 98.6%, respectively (Table S4). Similarly, absorbance maxima were lowered in reaction tubes containing PE24 extracts of irradiated *E. coli* by 10, 15, and 24 Gy which was translated to a reduction in total ADP-ribosylated product formed by 7%, 34%, and 48.92%, respectively (Table S5 and Fig. S15). Figure 3 evinces the chromatogram of reaction tubes post-exposure of *E. coli* BL21 (DE3) to low doses of gamma radiation. The chromatogram highlighted the absence of retention time shift and new peaks after NBAG peak at retention time (21–27 min).

**Fig. 4:**
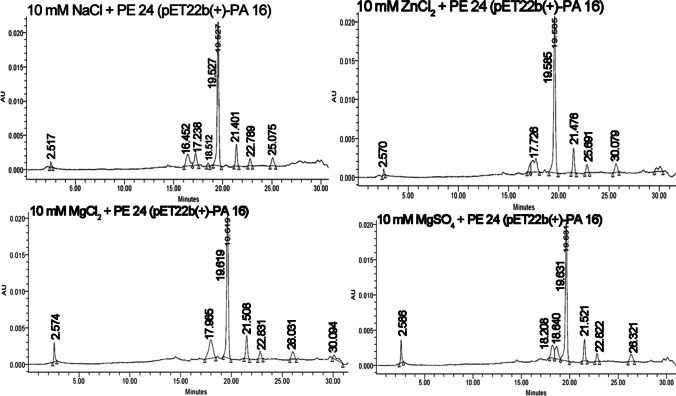
HPLC chromatogram for NBAG reaction tubes treated by different metal salts and PE24 extract retrieved from pET22b( +)-PA16 transformed *E. coli* BL21 (DE3)

On the other hand, the addition of metal salts to the reaction tube prior to the addition of PE24 moiety was also associated with eminent reduction and near abolishment in PE24 ADP-ribosylating activity (Tables S5 and S6). The addition of NaCl, ZnCl_2_, MgCl_2_, and MgSO_4_ was associated with a reduction in ADP-ribosylated NBAG by 85.7%, 82.09%, 80.8%, and 85.6%, respectively. The enzymatic activity of expressed PE24 was reduced to 0.09, 0.11, 0.12, and 0.09 U/h after the addition of NaCl, ZnCl_2_, MgCl_2,_ and MgSO_4_, respectively. Figure 4 depicts the reduction in intensity and the number of new peaks formed posts the addition of metal salts.

### Cytotoxicity of PE24 moiety on different cancer cell lines and impact of combing low-dose paclitaxel on IC50 value

**Fig. 5:**
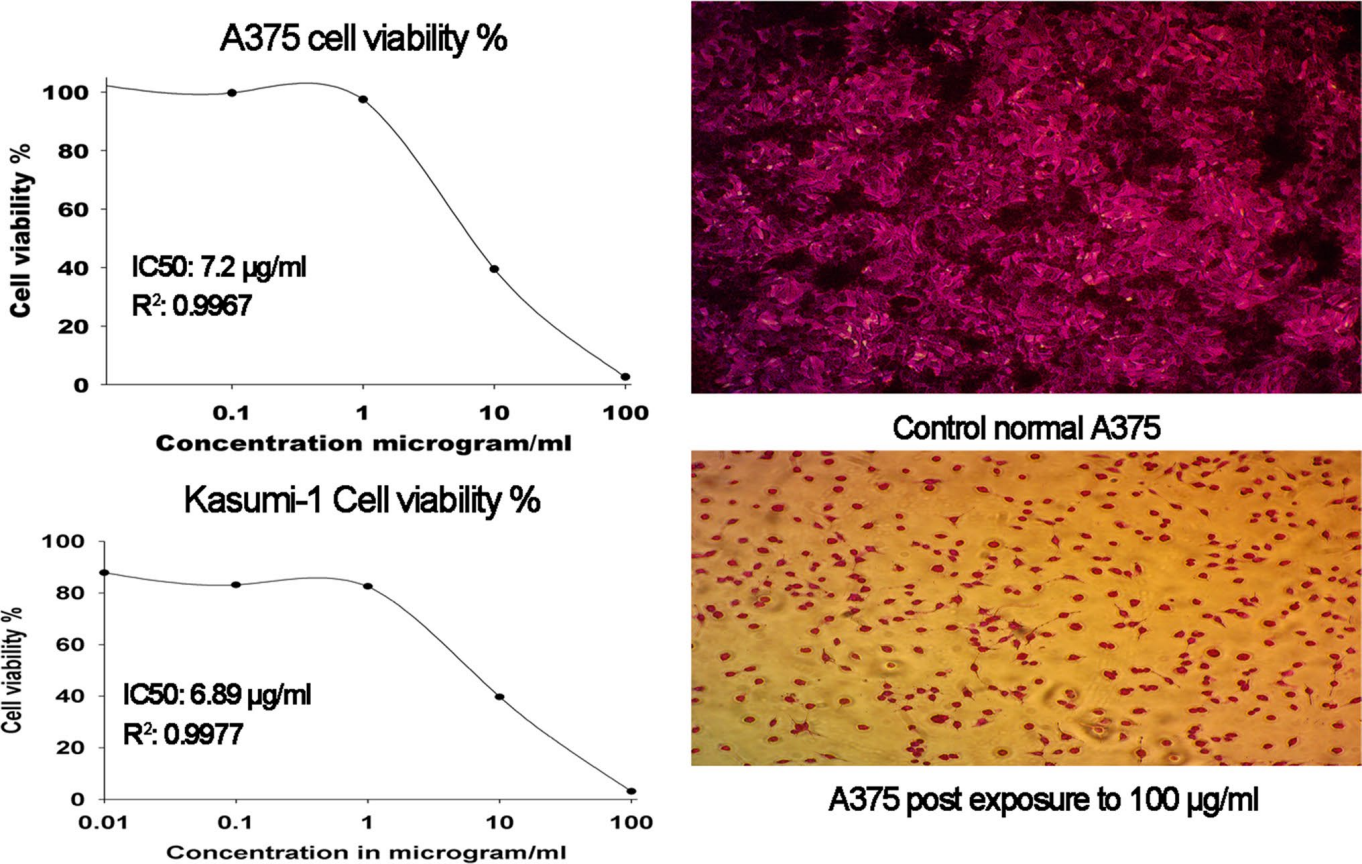
A375 and Kasumi-1 cell viability % plot post exposure to PE24 extract

**Fig. 6:**
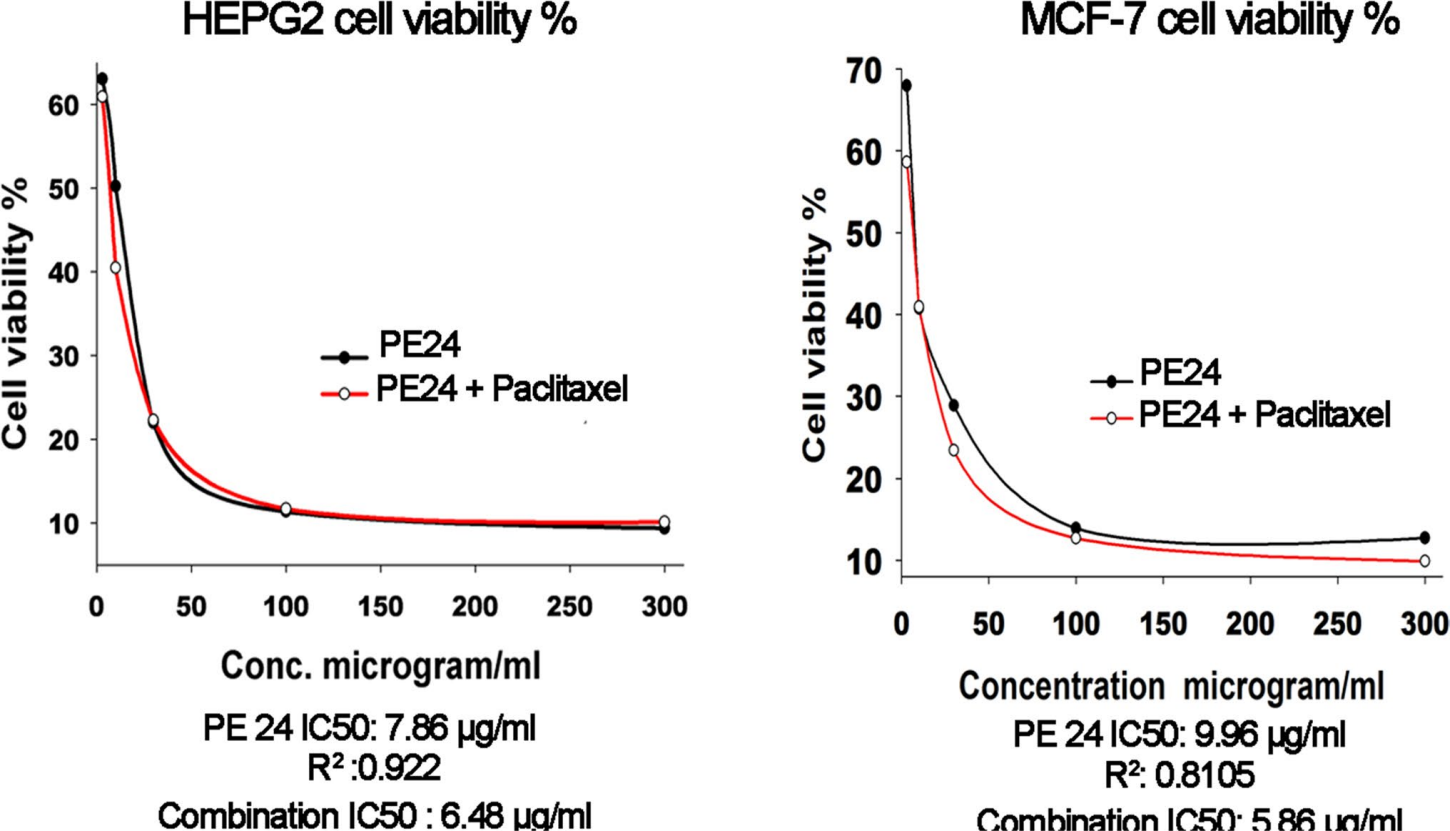
HEPG2 and MCF-7 cell viability % plot post exposure to PE24 extract alone or in combination with paclitaxel

The IC50 value of paclitaxel on HEPG2 and MCF-7 is < 10 μg/ml (less than 10 μM) with *R*^2^ value 0.6636 and 0.8113, respectively, whereas, doxorubicin scored IC50 value of < 0.01 μg/ml on Kasumi-1 and A375 cells with *R*^2^ value of 0.9963 and 0.9736, respectively (Figs. S16–S17, Tables S7–S10). On HEPG2 cells, PE24 moiety exhibited an eminent cytotoxic effect at 100 μg/ml scoring 88.6% (*P* < 0.05) and moderate cytotoxicity at 10 μg/ml of 49.79% (*P* < 0.05) (Table S7). On MCF-7 cells, PE24 moiety incurred significant cytotoxic effects of 86.1% and 59.24% at 100 μg/ml and 10 μg/ml, respectively (Table S8). Similar effects were observed on both A375 and Kasumi-1 cell lines, with prominent cytotoxicity at 100 μg/ml scoring 97.40% and 96.87% (*P* < 0.05), respectively. The moderate cytotoxic effect was noted by PE24 moiety on A375 and Kasumi-1 cell lines at 10 μg/ml recording 60.54% and 60.33% (*P* < 0.05), respectively (Fig. 5 and Tables S9–S10). The IC50 values for PE24 moiety on HEPG2, MCF-7, A375, and Kasumi-1 were 7.86, 9.96, 7.2, and 6.89 μg/ml, respectively. Interestingly, quick cytotoxicity screening of PE24 moiety on OEC: oral epithelial cell lines revealed a minor cytotoxic effect recording a cell viability percentage of 90.54% at 10 μg/ml (Table S10). Table 2 dissects IC50 values, *P* values, regression equation, and *R*^2^ value for PE24 moiety on the tested cell lines. Combining low-dose paclitaxel with PE24 moiety was associated with a significant reduction in IC50 values on both HEPG2 and MCF-7 cell lines. A 17.5% reduction in PE24 IC50 value was observed upon combining with 5 μg/ml pacilitaxel scoring 6.48 μg/ml on HEPG2 cells. On MCF-7 cell lines, a combination of paclitaxel with PE24 moiety reduced IC50 by 41.1% (Fig. 6 and Tables S7–S8).

**Table 2:** Regression analysis, Quick cytotoxicity screening on OEC cells an,d IC50 (μg/ml) on HEPG2, MCF-7, A375, and Kasumi-1 cell lines prospect-exposure PE24 moiety

Assay conducted	HEPG2	MCF-7	A375	Kasumi-1	OEC
	MTT		SRB	WST-1	
Cell viability at 10 μg/ml	50.21	40.75	39.45	39.67	90.93
*P* value	6.73 × 10^–9^	1.68 × 10^–9^	1.026 × 10^–9^	6.50 × 10^–11^	
Cell viability at 100 μg/ml	11.40	13.89	2.59	3.13	5.66
*P* value	1.11 × 10^–10^	7.45 × 10^–12^	4.41 × 10^–10^	4.33 × 10^–11^	
Regression equation	*y* = 69.07e^(−0.033X)^	*y* = 64.78e^(−0.0254X)^	*Y* = 103.1148e^(−0.0941X)^	*Y* = 86.81e^(−0.0773X)^	
*R*^2^ value	0.922	0.8105	0.9967	0.9977	
IC50 μg/ml	7.86	9.96	7.2	6.89

### Cytotoxicity of PE24 on gamma-irradiated HEPG2 cells and in combination with low-dose gamma radiation

**Fig. 7:**
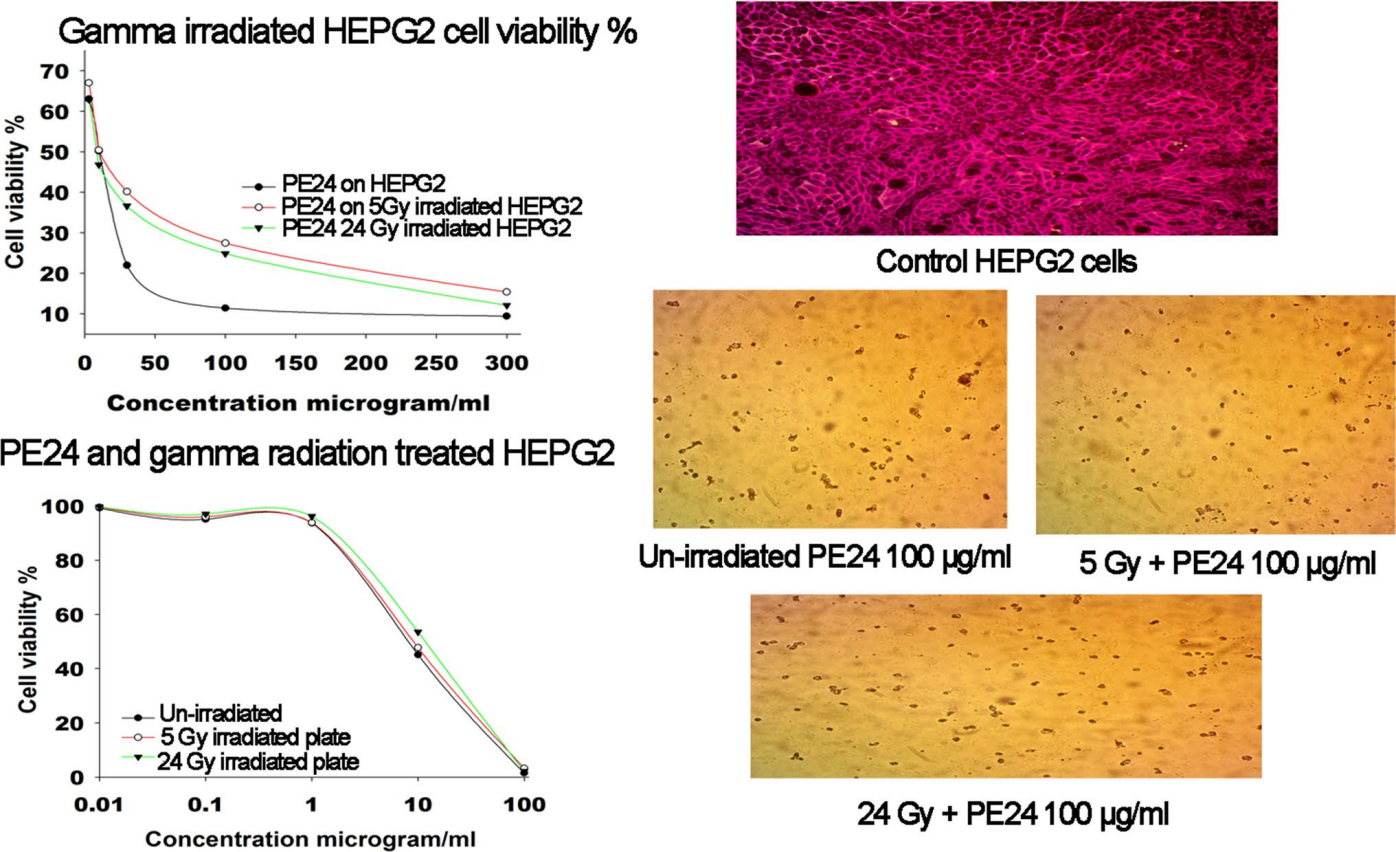
HEPG2 cell viability % plot post exposure to PE24 extract and low-dose gamma radiation

Treatment of HEPG2 cells by gamma ray doses, the low-dose 5 Gy and one shot 24 Gy were associated with an increase in IC50 values of PE24 moiety. Table 3 and Fig. 7 demonstrate the rise in IC50 value in both cases, PE24 on gamma ray–treated HEPG2 cells and coupling PE24 with low-dose gamma radiation. A 27.2% and 56% increase was recorded in PE24 IC50 value of gamma irradiated HEPG2 cells by 5 and 24 Gy, respectively (Table S11). In a similar manner, irradiating HEPG2 cells pre-exposed to PE24 moiety with 5 Gy and 24 Gy was associated witan h 8.3% and 26.3% increase in IC50 value, respectively (Table S12).

**Table 3:** Regression analysis and IC50 (μg/ml) value for PE24 moiety post-exposure to gamma-irradiated HEPG2 cells and in combination with low-dose gamma radiation

	Gamma-irradiated HEPG2 cells		Combination with low doses of gamma radiation on HEPG2 cells	
	5 Gy	24 Gy	5 Gy	24 Gy
Assay conducted	MTT		SRB	
Cell viability at 10 μg/ml	50.40	46.78	47.74	53.53
*P* value	6.94 × 10^–7^	9.27 × 10^–5^	4.36 × 10^–8^	3.59 × 10^–8^
Cell viability at 100 μg/ml	27.42	24.84	3.29	2.12
*P* value	1.07 × 10^–5^	1.29 × 10^–5^	2.02 × 10^–12^	2.91 × 10^–12^
Regression equation	Y = 58.09 e^(−0.0067X)^	Y = 55.0435 e^(−0.0078X)^	Y = 95.95e^(−0.0722X)^	Y = 99.73e^(−0.0614X)^
*R*^2^ value	0.9242	0.9345	0.9973	0.9981
IC50 μg/ml	10	12.28	9.38 μg/ml	10.94 μg/ml
Increase in IC50	2.14	4.42	0.72	2.28
Percent increase in IC50	27.2%	56%	8.3%	26.3%

Increase in IC50: PE24 IC50 after gamma irradiation-PE24 IC50 without radiation; percent increase in IC50%: increase in IC50/PE24 IC50 × 100 phosphate-buffered

## Discussion

In this study, catalytic domain Ib/III (PE24 moiety) retrieved from clinical *P. aeruginosa* isolates was reproduced inside pET22b( +) by *E. coli* BL21 (DE3). Following protein production, the ADP-ribosylating activity of PE24 moiety has been tested on nitrobenzylidene aminoguanidine (NBAG) and on in vitro cultured cell lines (OEC, HEPG2, MCF-7, A375: Human melanoma and Kasumi-1 cell lines). The study confirmed that expressed PE24 moiety was catalytically active and retained its ADP-ribosyl transferase activity post-expression by *E. coli* BL21 (DE3).

Firstly, the primers used during the PCR amplification reaction amplified *toxA* gene segment that encoded domain IB/III of *Pseudomonas aeruginosa* exotoxin A along with part from domain II encoding furin cleavage site. This segment has been previously identified as PE24 moiety (Kaplan et al. 2016) and it appeared around (~ 600–700 bp) in agarose gel electrophoresis. The primers also introduced *Nde*I and *Eco*R1 restriction sites to facilitate digestion by both enzymes and the generation of sticky ends for ligation with digested pET22b( +). The resultant PE24 amplicons harbored *Nde*I and *Eco*R1 restriction sites and pET22b( +) plasmid was extracted and purified from *E. coli* DH5α. Following the amplification reaction, restriction, and ligation reaction, the transformation of *E. coli* BL21 (DE3) by pET22b( +)-PA 16 and pET22b( +)-PA 22 constructs was performed and proved successful by colony PCR and the appearance of insert post digestion by *Nde*I and *Eco*R1. The expression of PE24 moiety was initiated by IPTG induction where approximately a 30 KDa protein migrated through acrylamide gel in the extracts retrieved from *E. coli* BL21 (DE3) transformed by pET22b( +)-PA16 and pET22b( +)-PA22 constructs only. This band was absent in extracts of *E. coli* transformed by empty pET22b( +) and non-transformed *E. coli* BL21 (DE3). It was reported by previously published work that PE24 moiety molecular weight alone was around 29–30 KDa (Kaplan et al. 2016; Wang et al. 2017; Liu et al. 2020). The appearance of ~ 30 KDa in SDS PAGE of protein extracts retrieved from pET22b( +)-PA 16/PA 22 harboring *E. coli* BL21 (DE3) suggests the expression of the protein.

Secondly, PE24 produced by *E. coli* BL21 (DE3) ADP-ribosylating ability was assessed as previously discussed (Soman et al. 1983; Morgan et al. 2021a, b). The NBAG dispersed in 0.1 N HCl peaked at *λ*_max_ 317 nm in UV absorbance spectrum (Soman et al. 1983; Morgan et al. 2021a, b). Higher wavelength (*λ*_max_) displacement in the UV spectrum to approximately 365–370 nm post-exposure of reaction tubes to PE24 extract indicated ADP-ribosylation of NBAG. This new *λ*_max_ is assigned to NBAG guanidine group deprotonation post attachment of ADP ribose sugar by PE24 moiety (Zhou et al. 2013). Soman et al. (1983) reported that a 0.1 unit difference in absorbance post-exposure of the reaction tube to enzymes is associated with the formation of 5 µmol/ml ADP-ribosylated NBAG. This indicated that the enzymatic activity of PE24 expressed in the unit (U) is equivalent to the amount of ADP ribosylated NBAG formed in micromolar per ml per hour (King and Campbell 1961; Soman et al. 1983). Furthermore, according to the IUB nomenclature committee, one unit of enzyme activity is equivalent to 1 μmol/min of the substrate formed per 1 ml (Karlson et al. 1979). The PE24 extracts retrieved from pET22b( +)-PA 16/22 transformed *E. coli*-produced ADP-ribosylated products of 0.482 and 0.542 μmol/ml/h. Furthermore, the reaction mixture was analyzed by FTIR spectroscopy and C^13^-NMR as elucidated in our results. The rise of a forked peak at 997–1000 cm^−1^ denoted the presence of phosphate group (PO_4_) in ADP-ribosylated product, whereas the shift and reduction in broad peak located at 2936–3020 cm^−1^ indicated the presence of OH from ribose sugar (Krüger et al. 2019). The 2160 cm^−1^ peak expounded the presence of C = N aromatic retrieved from ADP ribose sugar and the peaks located at 1512–1343 cm^−1^ belonged to NO_2_ and C–N retained from the original structure of nitrobenzylidene of NBAG (Moraes et al. 2008; Morgan et al. 2021a, b). These dissected structural changes indicated the hydrolysis of glycoside C–N of NAD^+^ and substitution of the guanidine group by ADP ribose sugar moiety in NBAG structure. The NBAG C^13^-NMR spectrum has been previously published in an additional file of the work conducted by Morgan et al. (2021a, b). The structural changes in NBAG NMR spectrum post-exposure to PE24 moiety confirmed its ADP-ribosylating activity. The rise of peaks at 61.5 and 59.5 ppm suggested the presence of RCH_2_OH which arise from the ADP ribose moiety. The bands at 45 and 124 ppm were assigned to RCH_2_NH_2_ and aromatic C, respectively. Bands appearing at 29.49, 22.76, and 40.18 ppm denoted the existence of RCO and RCH_2_ NH_2_, respectively, whereas the band located at 39.55 is the band of the DMSO-d6 solvent that has been used (Miwa et al. 1977; Chow et al. 2014). Both FTIR and C^13^-NMR confirm ADP-ribosylation of NBAG post-exposure to PE24 toxin extract.

ADP-ribosylated NBAG was also detected by HPLC as previously described (Morgan et al. 2021a, b). The NBAG peak usually appears at the retention time range of 9–13 min (Morgan et al. 2021a, b) taking in consideration the slight variation due to gradient elution (changes in methanol concentration against time). The prominent shift in NBAG peak and new peaks aroused around retention time 21 to 26 min suggested the formation of ADP-ribosylated products. The rise of peaks before the NBAG peak proposed the hydrolysis of NAD^+^ and breakage of glycosidic C–N bond by PE24 moiety releasing AMP, ADP ribose, and nicotinamide appearing at retention time range 13–17 min (Hachisuka et al. 2017; Petrova et al. 2017; Morgan et al. 2021a, b)**.** The PE24 extract retrieved from pET22b( +)-PA 16 transformed *E. coli* BL21 (DE3) exerted an enzymatic activity of 0.6 U/h and ADP-ribosylated NBAG by 16.4% whereas PE24 from pET22b( +)-PA 22 transformed *E. coli* BL21 (DE3) scored 1.08 U/h enzymatic activity and ADP-ribosylated NBAG by 34%. Wherefore, UV spectroscopy, IR, C^13^-NMR, and HPLC confirmed the ADP-ribosylating capability of produced PE24 confirming that expressed protein is catalytically active.

Thirdly, low-dose gamma ray irradiation of pET22b( +)-PA 16 containing *E. coli* BL21 (DE3) nearly abolished the ADP ribosyl transferase action of PE24 as elucidated by UV spectroscopy and HPLC. Reduction in ADP-ribosylating activity occurred in a dose-dependent manner. Irradiated plasmids are known for their low transformation efficacies and expression profiles. In a study conducted by Ishii et al. (2007) on the pEGFP plasmid that harbors *Eco*R1 sites as a model for extracellular DNA, DSBs that are prominent with increasing gamma radiation doses were prevalent among *Eco*R1 cloning sites of the plasmid. These breaks reduced the transformation efficacy and expression of the EGFP gene. PE24 moiety was cloned in between *Nde*I and *Eco*R1 site in pET22b( +) plasmid. Increasing the gamma radiation doses to 24 Gy might have incurred DSBs in the *Eco*R1 site which in turn damaged the genetic insert and thus reduced the expression and activity of the protein. Additionally, exposure to PE24 toxin extract from pET22b( +)/PA 16 transformed *E. coli* BL21 (DE3) to different metal salts resulting in prominent inhibition of ADP-ribosyltransferase activity. This was attributed to the conformational changes induced by the metal ions (Li et al. 2015; Özaslan et al. 2017).

To monitor in vitro ADP-ribosyl transferase cytotoxicity of PE24 on solid and hematological tumors, different cancer cell lines were used. The adherent cells that mimic solid carcinomas models were HEPG2, MCF-7, and A375, while, Kasumi-1 cell suspension resembled the hematological carcinoma model. Multiple in vitro cytotoxicity assays were conducted in this study for technical reasons. First, Kasumi-1 cell line was obtained in cell suspension form and the best way to assess cell suspension cytotoxicity was through conductance of MTT or WST-1 cytotoxicity assay as previously reported since SRB assay requires cell fixation and works mainly with adherent cells (Orellana and Kasinski 2016). WST-1 assay was preferred with Kasumi-1 cell lines than MTT since it required lower incubation time, and produced better linearity and *R*^2^ value (Table 2) as previously reported (Mitkevich et al. 2011; López et al. 2020). Secondly, both MTT and SRB assays were conducted on adherent cell lines; however, SRB assays produced good linearity and *R*^2^ value than MTT, especially in the case of A375 cells (Tables 2, 3 and Tables S7–S9). This was previously reported among in vitro cultivated A375 (Papadimitriou et al. 2019). The SRB assay was also performed as it is more relatable for PE24 ADP-ribosyl transferase cytotoxicity since SRB dye binds to protein residues fixed by trichloroacetic acid and did not depend on the metabolic activity of affected cells (Orellana and Kasinski 2016; Papadimitriou et al. 2019). Wherefore, it was more sensible to conduct both SRB and MTT assays upon monitoring PE24 ADP-ribosyl transferase cytotoxicity during in vitro laboratory assays. The choice of HEPG2 cells to assess the impact of gamma irradiation and paclitaxel on in vitro ADP-ribosyl transferase activity of PE24 moiety was based on the fact that HEPG2 cells are known for their high chemotherapeutic and radiotherapy resistance. Recent research was dedicated to generating ITs harnessing PE24 to combat chemo/radio-resistant HEPG2 cells (Chen et al. 2022; Heiat et al. 2021). For this reason, HEPG2 cells were used while exploring the impact of gamma radiation and paclitaxel on PE24 moiety.

Interestingly, PE24 moiety retrieved extract exhibited prominent cytotoxic effects on the adherent cell lines, HEPG2, MCF-7, A375, and Kasumi-1 cell suspension as depicted by different cytotoxic assays. The IC50 values of PE24 moiety recorded were below 10 μg/ml with *R*^2^ values ranging from 0.8105 to 0.9977. This means that PE24 moiety exhibited a good inhibitory effect on cancer cell lines (Indrayanto et al. 2021). The anticancer activity of PE24 moiety is probably attributed to its ADP-ribosylating activity (Kaplan et al. 2016; Wang et al. 2017). It was also noted that 10 μg/ml of PE24 extract showed ~ 90% cell viability on normal OEC cells. This indicates that the PE24 moiety incurs less cytotoxic effects on the normal cells as previously reported (Havaei et al. 2021).

To explore the effect of paclitaxel on the ADP-ribosyl transferase activity of PE24 moiety, a combination of low-dose paclitaxel (5 μg/ml) with a different dilution of PE24 moiety was prepared and IC50 value was recorded. The addition of low-dose paclitaxel (5 μg/ml) to PE24 was associated with a prominent reduction in IC50 value by 17.5% in the case of HEPG2 and 41.1% for MCF-7. The selection of paclitaxel dosage (5 μg/ml) was related to scored IC50 value by paclitaxel on HEPG2 and MCF-7 cells (< 10 μg/ml, < 10 μM) using MTT assay (Fig. S16 and Tables S7–S8). Both cell lines exhibited non-linear cytotoxicity and viability patterns post-exposure to paclitaxel at different concentrations (Tables S7–S8 and Fig. S16). This non-linear effect could be assigned to the fact that paclitaxel’s anti-proliferative and cytotoxic effects vary in rapidly growing in vitro cultured cell lines. Nevertheless, their cytotoxic effects are better explored throughout short time intervals (< 20 h) (Risinger et al. 2015). Alexandre et al. recorded the IC50 of paclitaxel alone on A549: lung carcinoma cell line at 24 h. of 5 μM and it was lower only when the initial cell count was less than 10^4^ (Alexandre et al. 2006). Furthermore, variation in IC50 value scored by MTT assay in vitro studies has also been reported and rarely given a consistent IC50 value for anticancer agents over different cancer cell lines. For instance, cisplatin recorded an IC50 value of 2 to 40 μM on SKOV-3 cells by 24 h MTT assay (He et al. 2016). Based on the recorded effects of paclitaxel on in vitro cultivated HEPG2 and MCF-7, the paclitaxel was used at 5 μg/ml which is equivalent to ~ 5 μM during the assessment PE24 moiety combination therapy. Combining paclitaxel with PE24 did not reduce its ADP-ribosyl transferase cytotoxicity, conversely, it reduced IC50 value suggesting that the amide group of tetracyclic diterpenoid paclitaxel structure did not compete with NAD^+^ for PE24 active site. This observation was in line with previously published reports where these synergistic effects might be attributed to enhanced cellular uptake of PE24 moiety by cancer cells post-exposure to paclitaxel (Zhang et al. 2010; Müller et al. 2017).

The gamma irradiation doses selected to assess the impact of gamma radiation on the ADP-ribosyl transferase activity of PE24 were 5 and 24 Gy. The 24 Gy radiation dosage is best known as one-shot stereotactic single-dose radiotherapy (SDRT) that is commonly used with oligometastatic cancer patients suffering from brain and prostate carcinoma (Zilli et al. 2018; Greco et al. 2021; Palmer et al. 2020). Moreover, it has been concurrently administered with monoclonal antibodies such as ranibizumab during the treatment of neovascular age-related macular degeneration (Jackson et al. 2013). The 24 Gy SDRT was also associated with better efficacy, tolerability, and high control rate among spinal carcinoma metastases patients (Gong et al. 2019). The lower gamma dose of 5 Gy resembled the fractionated stereostatic radiotherapy where a 5 Gy dose is delivered in 2 to 4 fractions (sometimes 5) over a period of 2 to 4 days. The 5 Gy fractionation dosage has been used in several carcinomas including non-small cell lung, colorectal, breast, metastatic brain, and hematological carcinomas and proved to be successful in improving overall survival rate (Kim et al. 2016). Antagonistic effects were observed among HEPG2 cancer cell lines pre-exposed to low-dose 5 Gy and one shot 24 Gy treated by PE24 moiety and combining PE24 moiety with both dosages on HEPG2 cells. The IC50 values of PE24 moiety were increased in both treatment scenarios. A 27.2% and 56% increase in PE24 IC50 value was reported among HEPG2 cells pre-exposed to gamma radiation at 5 Gy and one shot at 24 Gy, respectively. Irradiating HEPG2 cells post-exposure to PE24 was associated with an elevation in IC50 values by 8.3% and 26.3% at 5 and 24 Gy, respectively. This result contradict previously published reports that confirmed the enhancement of PE-IT activity by gamma radiation for its inhibitory effect on cancer cell antigen shedding and radiation-induced mutation in diphthamaide residue that renders elongation factor 2 more susceptible to PE toxin (Dieffenbach and Pastan 2020). This effect could be explained in regards to both HEPG2 cells and ADP-ribosyl transferase activity of PE24 toxin moiety. As previously elucidated, low-dose gamma ray–irradiated HEPG2 exhibited stemness and cellular rounding (Ghisolfi et al. 2012). Cancer cell stemness enhances their differentiation and alters their interaction with the surrounding microenvironment, affecting their quiescence, proliferation, and regeneration and rendering them more resistant to external treatment (Aponte and Caicedo 2017). This explains the rise in IC50 of PE24 moiety on gamma-irradiated HEPG2 cells. Upon coupling gamma radiation with PE24 moiety, the IC50 values were increased, however, the increase was lower than exhibited by gamma-irradiated HEPG2 cells. This could be attributed to the fact that gamma rays could have incurred local conformation changes in the PE24 active site that had resulted in a partial reduction of ADP-ribosyl transferase activity. Conformational changes in protein structures post-exposure to low doses of gamma rays (1–10 Gy) is common and were associated with denaturing effects previously elucidated on different proteins in several reports (Morgan et al. 2021a, b; Santos et al. 2020; Mahmoud et al. 2011). In conclusion, PE24 moiety was successfully cloned and expressed by pET22b( +) inside *E. coli* BL21 (DE3). The expressed protein retained its ADP-ribosyl transferase activity on NBAG as recorded by UV, IR spectroscopy, C13-NMR, and HPLC confirming the expression of active peptide. Irradiating *E. coli* BL21 (DE3) resulted in the reduction of the expression profile and activity of the PE24 moiety. In similar manners, exposure to metal salts drastically reduced its ADP-ribosylating activity suggesting caution upon using buffers with divalent/monovalent metal ions. PE24 extract was cytotoxic on adherent cell lines HEPG2, MCF-7, and A375 and Kasumi-1 cell suspension at IC50 < 10 μg/ml and showed tolerable cell viability on normal OEC cells at 10 μg/ml. This suggested its efficacy and tolerability. Combination with low-dose paclitaxel enhanced its cytotoxic activity whereas gamma irradiation of cells before and after exposure to PE24 moiety lowered its efficacy. This might be alerting upon combining immunotoxin therapy with radiotherapy for the treatment of hepatocellular carcinomas.

## Supplementary information

Below is the link to the electronic supplementary material.

Supplementary file1 (PDF 1769 KB)

## Data Availability

The authors declare that the data supporting the findings of this study are available within the article and its supplementary information file. The 16S ribosomal RNA of *P. aeruginosa* PA16 and PA22 isolates were deposited in the NCBI GenBank database under the accession codes, MZ713410.1, and MZ713405.1, respectively. The sequence for cloned PE 24 moiety has been submitted into the NCBI GenBank database under the accession code, OP889245.
