# Correction to: Gamma radiation coupled ADP‑ribosyl transferase activity of *Pseudomonas aeruginosa* PE24 moiety

**DOI:** 10.1007/s00253-023-12455-x

**Published:** 2023-03-07

**Authors:** Radwa N. Morgan, Sarra E. Saleh, Hala A. Farrag, Khaled M. Aboshanab

**Affiliations:** 1 National Centre for Radiation Research and Technology (NCRRT), Drug Radiation Research Department, Egyptian Atomic Energy Authority (EAEA), Ahmed El‑Zomor Street, Nasr City, 11787 Cairo Egypt; 2 Microbiology and Immunology Department, Faculty of Pharmacy, Ain Shams University, African Union Organization Street, Abbassia, 11566 Cairo Egypt

**Correction to: Applied Microbiology and Biotechnology**

**https://doi.org/10.1007/s00253-023-12401-x**

The original published version of this paper contained some mistakes in the equations.

On page 6 of the proof, two equations at the end of the second paragraph of “Cytotoxicity of PE24 moiety on HEPG2 and MCF‑7 cell lines alone and in combination with paclitaxel by MTT cell proliferation and cytotoxicity test” section should be corrected as:

$$HEPG2\;or\;MCF7\;\boldsymbol c\boldsymbol e\boldsymbol l\boldsymbol l\boldsymbol\;\boldsymbol v\boldsymbol i\boldsymbol a\boldsymbol b\boldsymbol i\boldsymbol l\boldsymbol i\boldsymbol t\boldsymbol y\;\%=\frac{\mathrm{PE}24\;\mathrm{Treated}\;\left(\mathrm{HEPG}2\;\mathrm{Or}\;\mathrm{MCF}7\right)\;\mathrm{cells}\;\mathrm{absorbance}}{\mathrm{untreated}\;\left(\mathrm{HEPG}2\;\mathrm{or}\;\mathrm{MCF}7\right)\;\mathrm c\mathrm e\mathrm l\mathrm l\mathrm s\;\mathrm a\mathrm b\mathrm s\mathrm o\mathrm r\mathrm b\mathrm a\mathrm n\mathrm c\mathrm e\;(\mathrm{control})}\times100\\$$

$$HEPG2\;or\;MCF7\;\boldsymbol c\boldsymbol e\boldsymbol l\boldsymbol l\boldsymbol\;\boldsymbol c\boldsymbol y\boldsymbol t\boldsymbol o\boldsymbol t\boldsymbol o\boldsymbol x\boldsymbol i\boldsymbol c\boldsymbol i\boldsymbol t\boldsymbol y\;\%\;=\;100\;-\;viability\;\%$$

On page 7of the proof, the equation at the end of the paragraph of “Cytotoxicity of PE24 moiety on A375: human melanoma cells by sulforhodamine B assay (SRB)” section should be corrected as:

$$A375\;\boldsymbol c\boldsymbol e\boldsymbol l\boldsymbol l\boldsymbol\;\boldsymbol v\boldsymbol i\boldsymbol a\boldsymbol b\boldsymbol i\boldsymbol l\boldsymbol i\boldsymbol t\boldsymbol y\;\%=\frac{\mathrm{PE}24\;\mathrm{Treated}\;\mathrm A375\;\mathrm{cells}\;\mathrm{absorbance}}{\mathrm{untreated}\;\mathrm A375\;\mathrm{cells}\;\mathrm{absorbance}\;(\mathrm{Control})}\times100$$

Still on page 7 of the proof, the equation at the end of the paragraph of “Cytotoxicity of PE24 moiety on OEC: normal oral epithelial cells and Kasumi‑1: acute monocytic leukemia (AML) cells using WST‑1 cytotoxicity assay” section should be corrected as:

$$OEC\;or\;Kasumi1\;\boldsymbol c\boldsymbol e\boldsymbol l\boldsymbol l\boldsymbol\;\boldsymbol v\boldsymbol i\boldsymbol a\boldsymbol b\boldsymbol i\boldsymbol l\boldsymbol i\boldsymbol t\boldsymbol y\;\%=\frac{\mathrm{PE}24\;\mathrm{Treated}\;\left(\mathrm{OEC}\;\mathrm{or}\;\mathrm{Kasumi}1\right)\;\mathrm{cells}\;\mathrm{absorbance}}{\mathrm{untreated}\;\left(\mathrm{OEC}\;\mathrm{or}\;\mathrm{Kasumi}1\right)\;\mathrm{cells}\;\mathrm{absorbance}\;(\mathrm{control})}\times100$$

This is being corrected in this publication.

