# Supplementary material for: Gamma radiation coupled ADP-ribosyl transferase activity of Pseudomonas aeruginosa PE24 moiety
Source: Appl Microbiol Biotechnol. 2023 Feb 18;107(5-6):1765–84. doi: 10.1007/s00253-023-12401-x (PMC10006270; doi:10.1007/s00253-023-12401-x)
Supplement: Supplementary file 1 — Supplementary file1 (PDF 1769 KB) [file 253_2023_12401_MOESM1_ESM.pdf]

Gamma radiation coupled ADP-ribosyl transferase activity of *Pseudomonas aeruginosa* PE24 moiety

Radwa N. Morgan[1], Sarra E. Saleh[2], Hala A. Farrag[1], Khaled M. Aboshanab[2*],

[1]National Centre for Radiation Research and Technology (NCRRT), Drug Radiation Research Department, Egyptian Atomic Energy Authority (EAEA), Ahmed El-Zomor Street, Nasr city, Cairo, 11787, Egypt

[2] Microbiology and Immunology Department, Faculty of Pharmacy, Ain Shams University, African union organization Street, Abbassia, Cairo, 11566, Egypt

Correspondence: Khaled M. Aboshanab (Ph.D.)**,** Professor of Microbiology and Immunology and Vice Dean of Graduate Studies, Faculty of Pharmacy, Ain Shams University**,** Cairo, Egypt.

E-mail: aboshanab2012@pharma.asu.edu.eg,

Tel: (202)28429040

Mobile: (002)01007582620

Fax: (202)24051107

https://orchid.org/0000-0002-7608-850X

Authors e-mails

Radwa N. Morgan: radwa_morgan@hotmail.com

Sarra E. Saleh: sarradeif@pharma.asu.edu.eg

Hala A. Farrag: hala_farrag_24@hotmail.com

Material and Method

1. DNA extraction technique using Thermofisher DNA purification kit (K0721, Massachusetts, USA):

An overnight bacterial broth of PA 16 and PA 22 (OD 0.8) was transferred to sterile Eppendorf and centrifuged for 10 min at 7000 xg followed by supernatant decantation. The precipitated pellet was dissolved in kit digestion solution with proteinase K, and incubated at 56°C for 30 min with occasional vortexing. The heated mixture was then supplemented with RNase A and brooded at room temperature (25°C) for 10 min. The lysis buffer and 50% ethanol were added to the mixture and transferred to kit purification columns. The purification columns were centrifuged at 7000, 8000, and 12000 xg for 1 to 3 min using kit washing buffers I and II, respectively as prescribed in the kit's guidelines. A final centrifugation round was performed at 8000 xg for 1 min to elute genomic DNA with kit elution buffer.

2. DNA concentration technique using Zymo Research DNA clean and concentrate TM-25 (DCC-25):

The PCR product was mixed with the kit binding buffer at a ratio of 5:1 (binding buffer: PCR reaction volume). The mixture was then transferred to zymospin columns and the amplicon was eluted after multiple centrifugation rounds at 11000 xg for 30 sec using the kit DNA washing buffers.

3. pET22b(+) plasmid extraction by Zymogen research Zyppy plasmid

E. coli DH5α bacterial broth was incubated with the blue 7x lysis buffer for 2 minutes at room temperature (25oC) followed by alkaline neutralization with yellow cold neutralization buffer. The sediment formed was sloughed off by 4 min centrifugation at 15000 xg and the supernatant was poured into Zyppy purification columns provided in the kit. The plasmid was eluted following multiple rounds of centrifugations using Zyppy end-wash and washing (I and II) buffers.

4. pET22b(+) plasmid purification using GeneJet gel extraction kit K0691

To purify excised pET22b(+) plasmid band, the binding buffer was used according to the ratio 1:1 (weight of gel in mg: microliters of binding buffers). The gel/binding buffer mixture was melted at 56°C for 10 min then the solution was transferred to purifying columns. The plasmid was eluted from the gel after several centrifugation rounds at 11000 xg for 1 minute using kit washing and elution buffer.

Figures

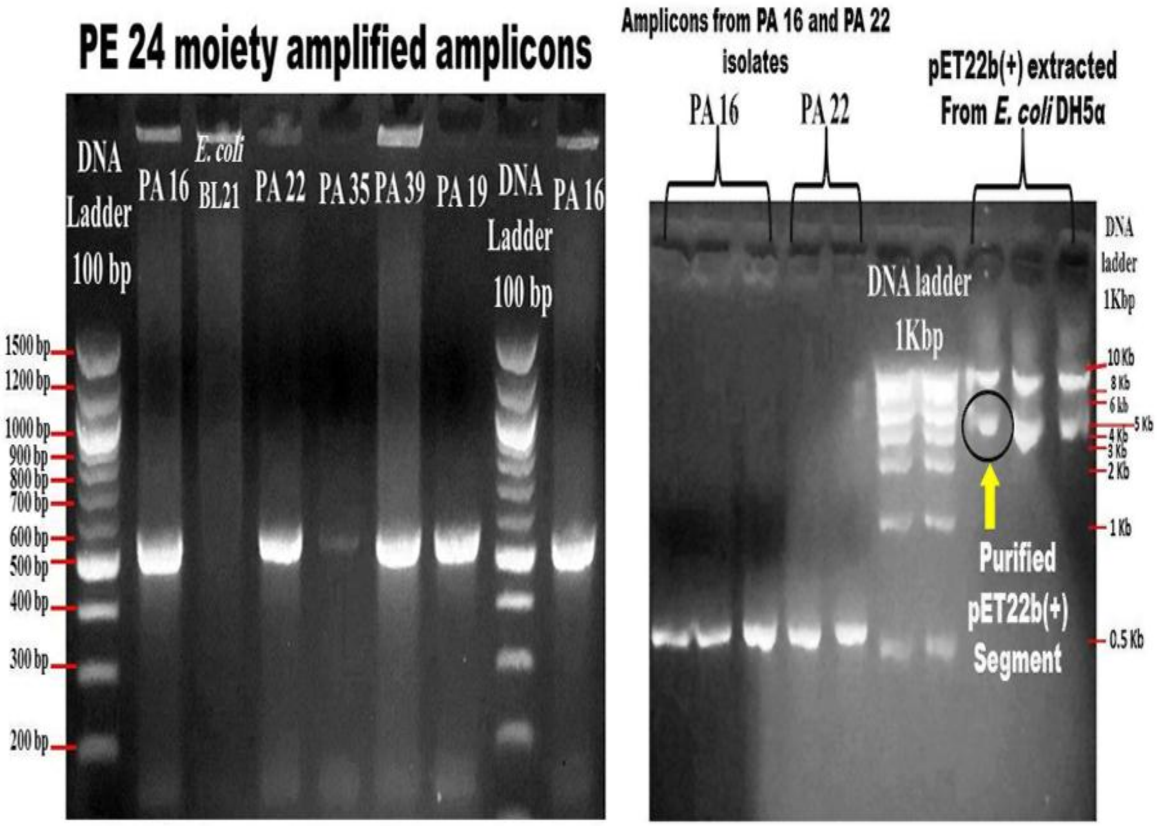

Fig. S1 Electrophoresed PE24 amplicons from DNA of different *P. aeruginosa* isolates and pET22b(+) extract retrieved from *E. coli* DH5α.

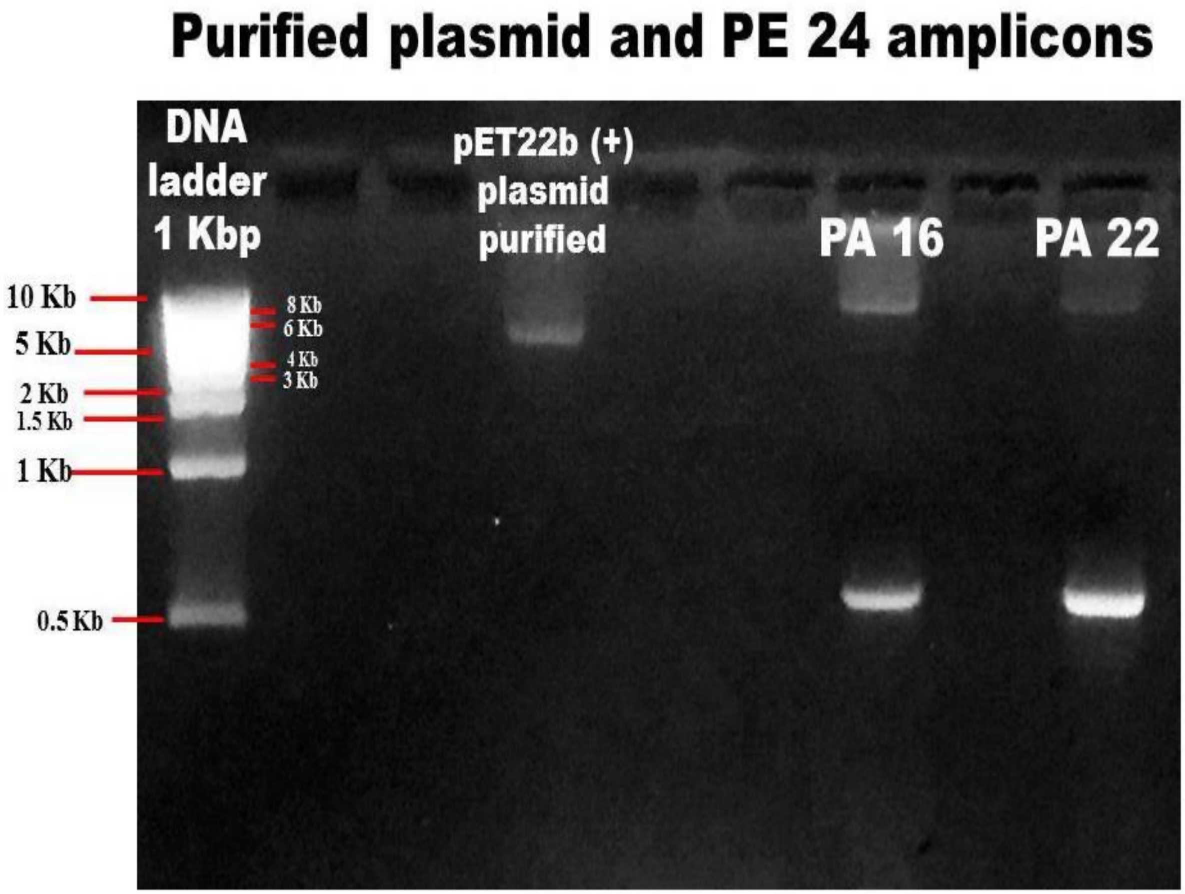

Fig. S2 Purified pET22b(+) 5000 Kbp band and PE 24 amplicons retrieved from PA16 and PA22 isolates.

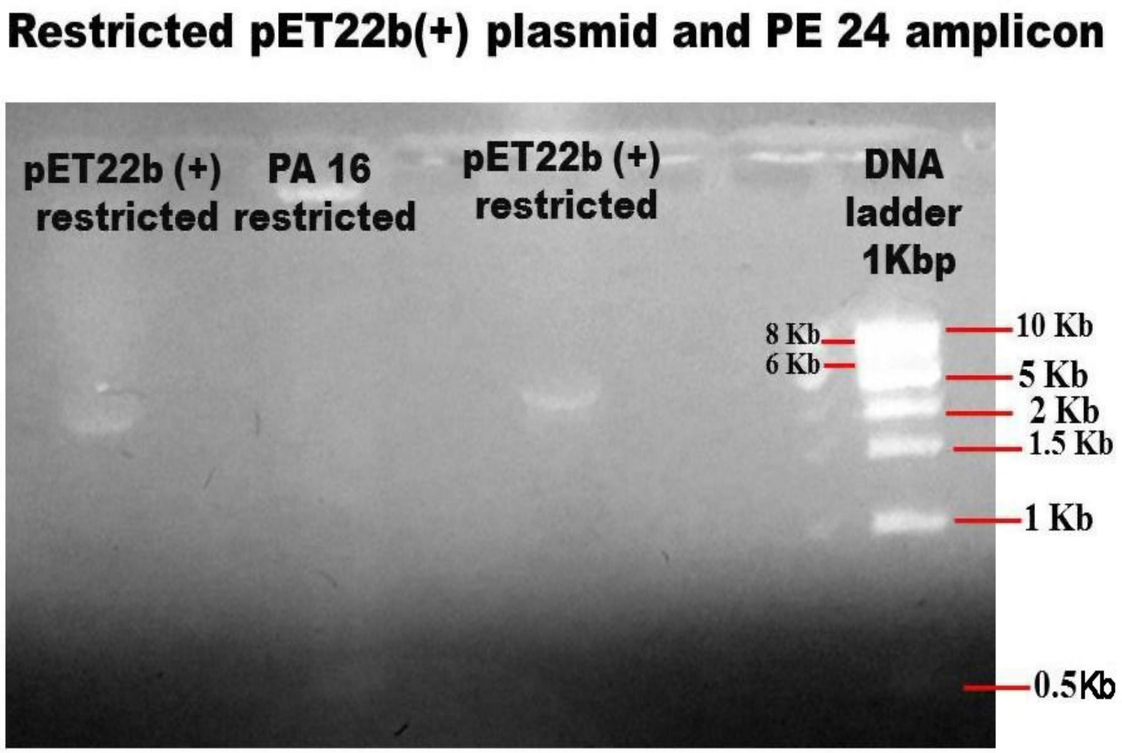

Fig. S3 Electrophoresed restricted pET22b(+) and PE 24 amplicon after exposure to *Nde*I and *Eco*R1 enzymes visualized after mixing with loading dyes.

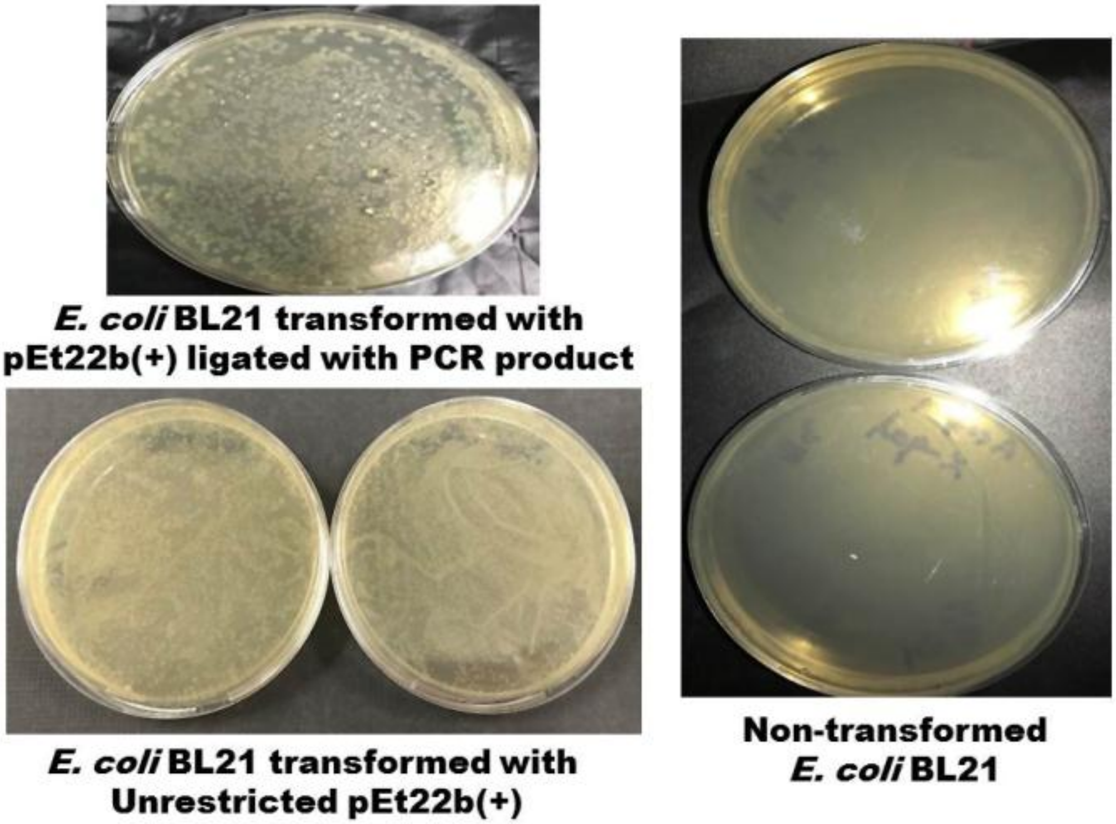

Fig. S4 LB agar plates containing 100 µg/ml ampicillin showing *E. coli* BL21 (DE3) growth after transformation with empty pET22b(+) and ligated pET22b(+) and non-transformed *E. coli* BL21(DE3).

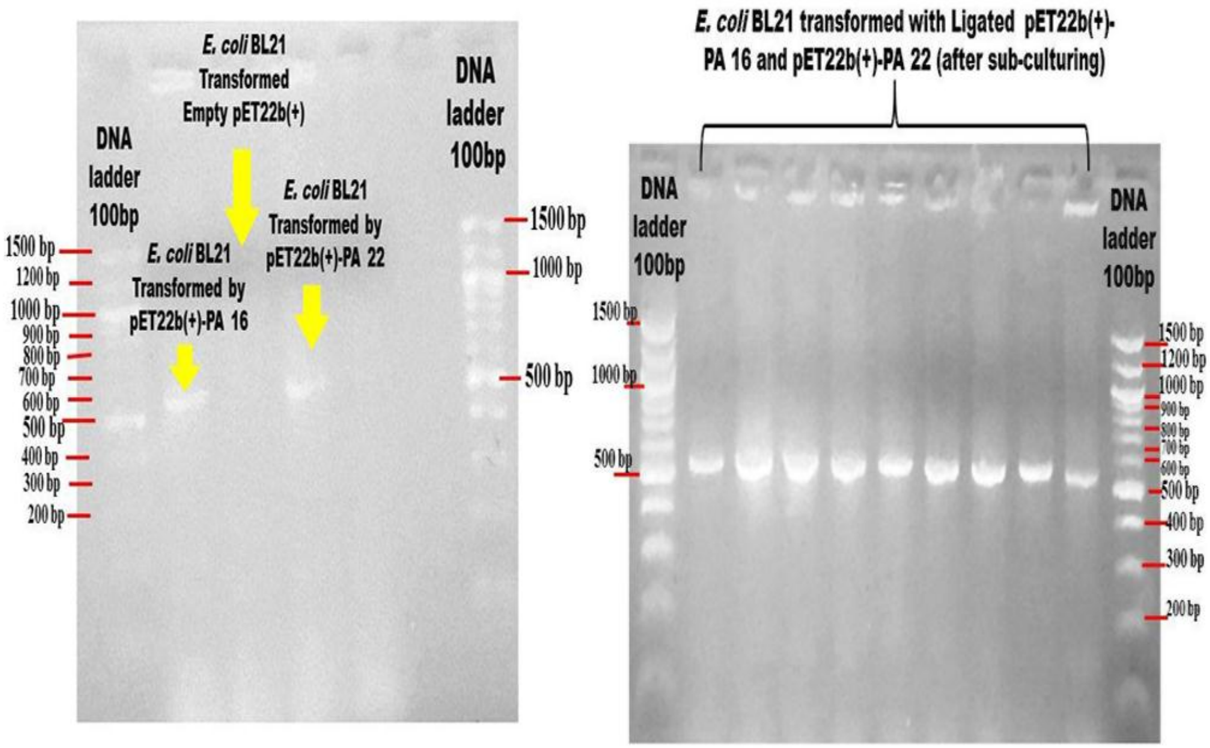

Fig. S5 PE24 amplicons electrophoresed from colony PCR post transformation of *E. coli* BL21 (DE3) by pET22b(+)-PA16/PA22 constructs.

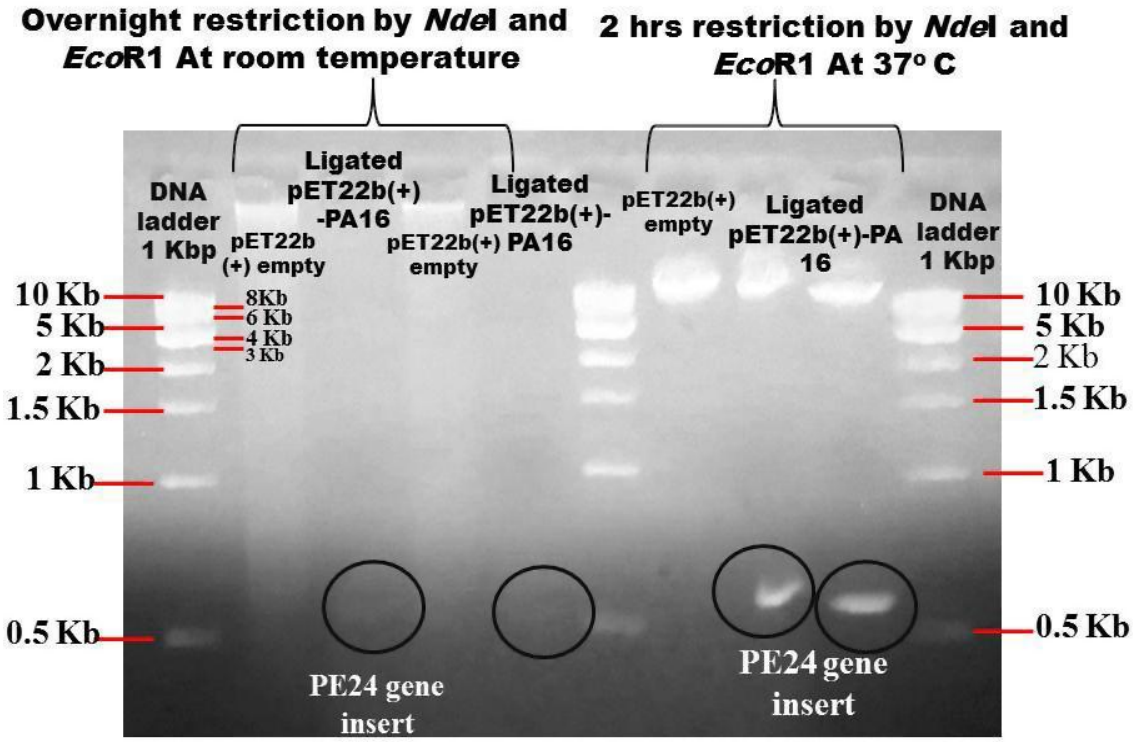

Fig. S6 Electrophoresed restriction products of empty pET22b(+) and ligated pET22b(+)-PA16

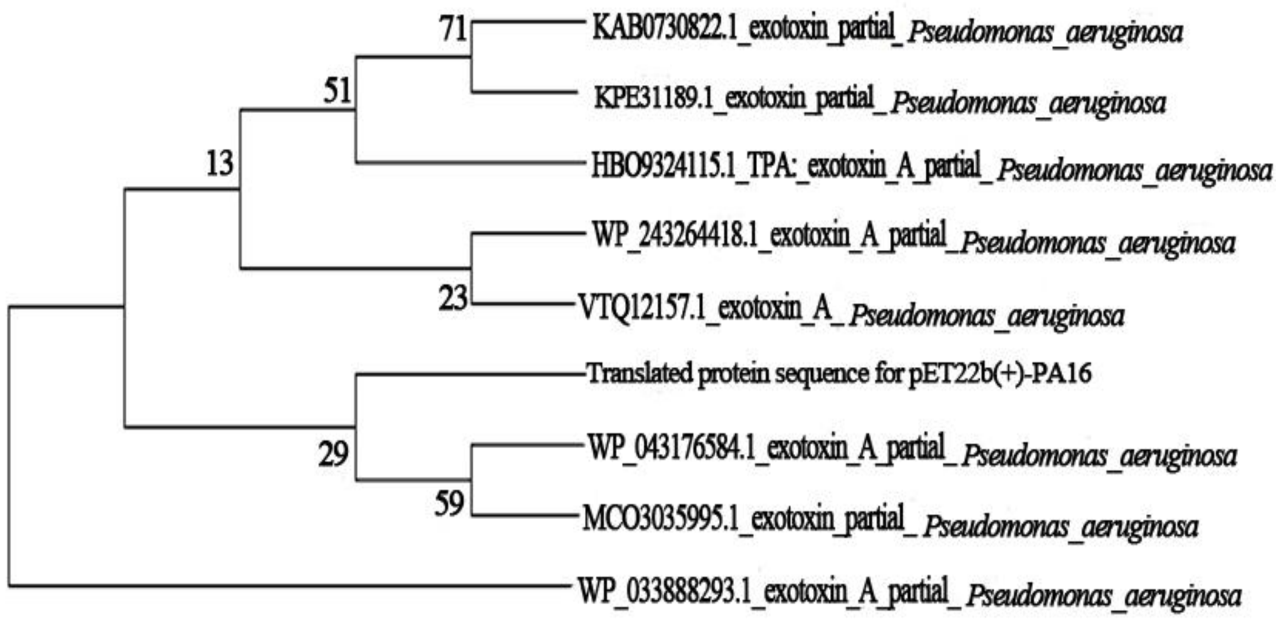

Fig. S7 Protein Alignment bootstrap tree.

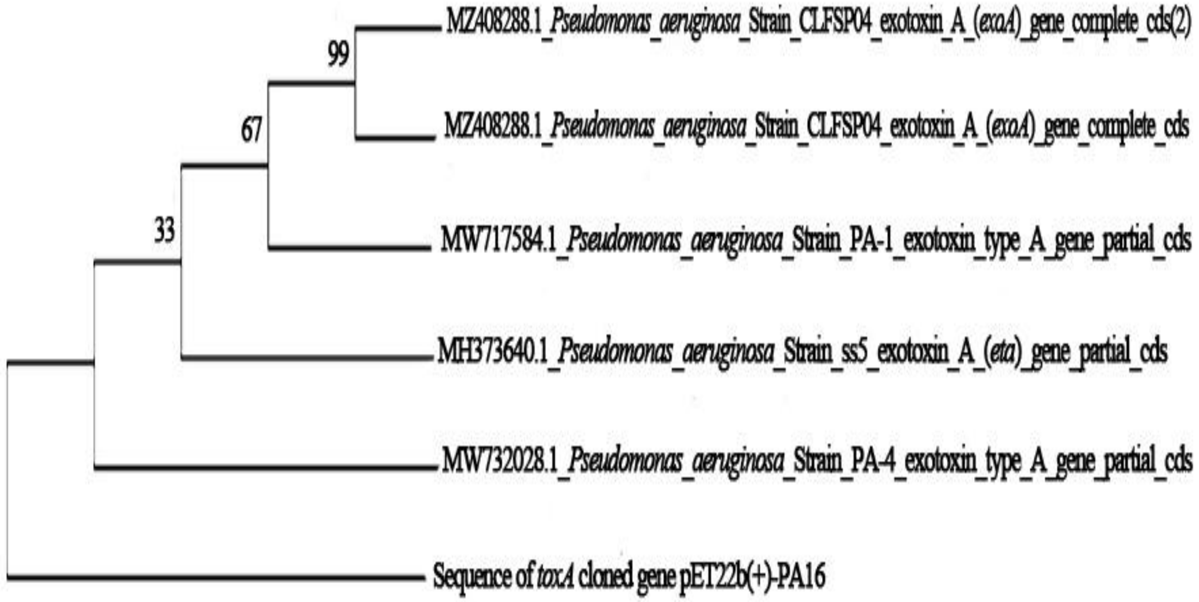

Fig. S8 Nucleotide Sequence Alignment bootstrap tree.

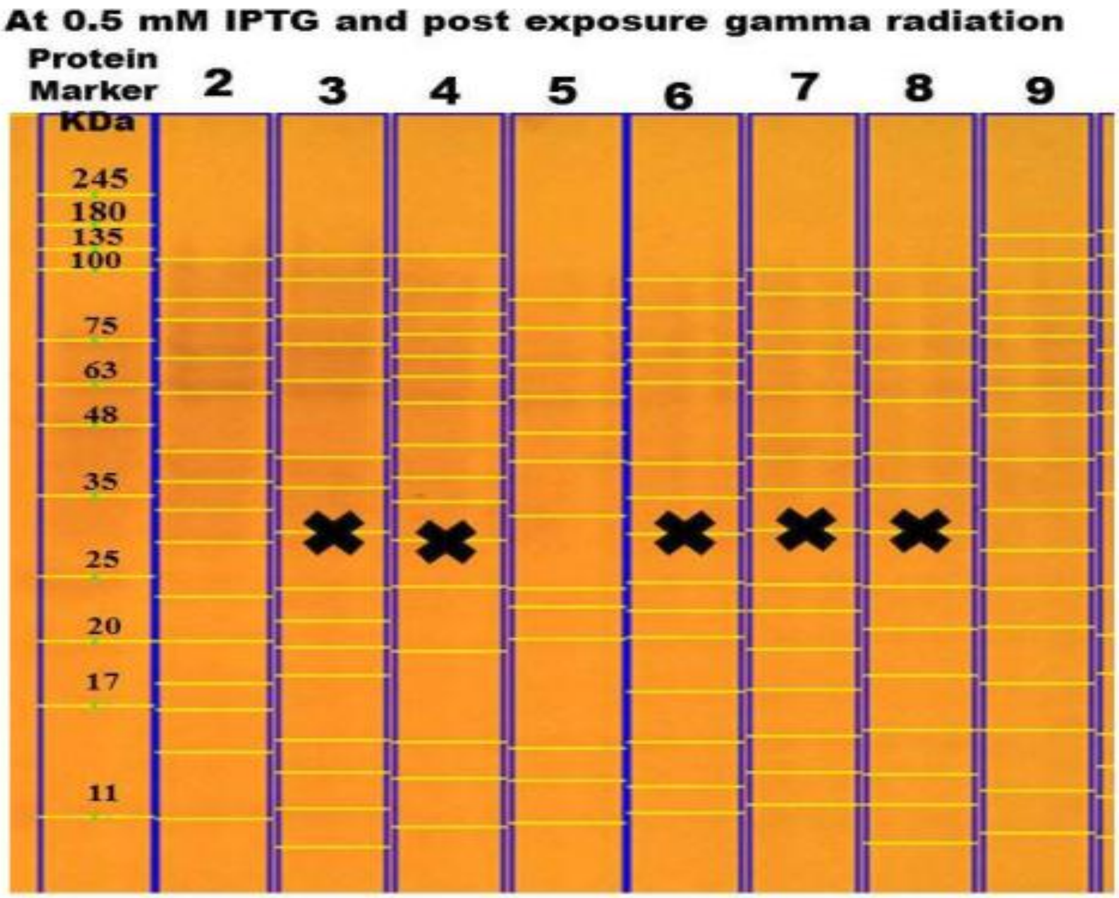

Fig. S9 SDS PAGE analysis for protein extracts retrieved from 0.5 mM IPTG induced *E. coli* BL21 (DE3) . 1: Protein Marker 2: *E. coli* BL21 (DE3) transformed by empty pET22b(+); 3: *E. coli* BL21 (DE3) transformed by pET22b(+)-PA16; 4: *E. coli* BL21 (DE3) transformed by pET22b(+)-PA 22; 5: *E. coli* BL21 (DE3) non-transformed; 6: 5Gy irradiated pET22b(+)-PA 16 transformed *E. coli* BL21 (DE3) ; 7: 10Gy irradiated pET22b(+)-PA 16 transformed *E. coli* BL21 (DE3) ; 8: 15 Gy irradiated pET22b(+)-PA 16 transformed *E. coli* BL21 (DE3) ; 9: 24Gy irradiated pET22b(+)-PA 16 transformed *E. coli* BL21 (DE3) .

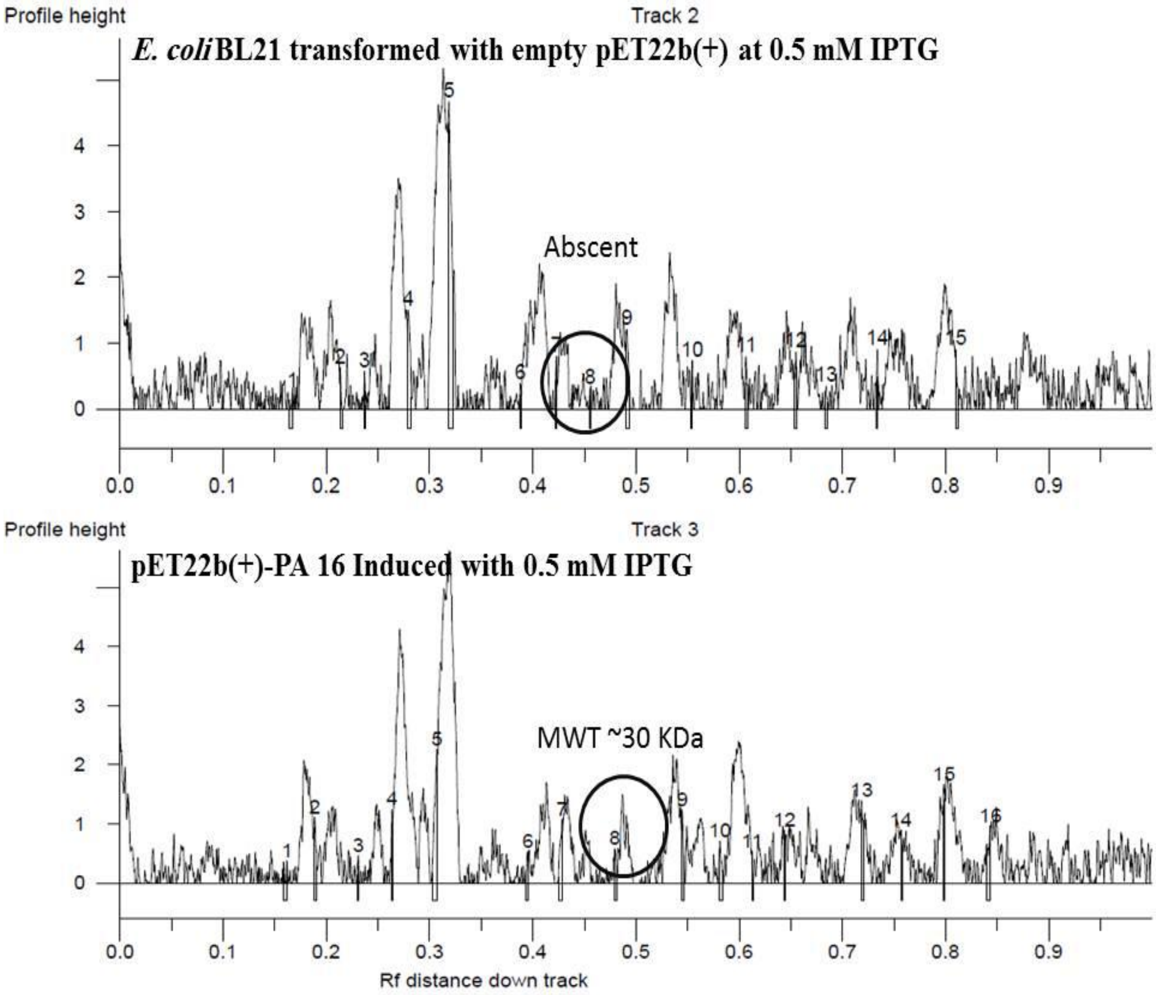

Fig. S10 Rf distance down track plot for electrophoresed protein retrieved from 0.5 mM IPTG induced pET22b(+)-PA16 transformed *E. coli* BL21 (DE3) and empty pET22b(+) transformed *E. coli* BL21(DE3).

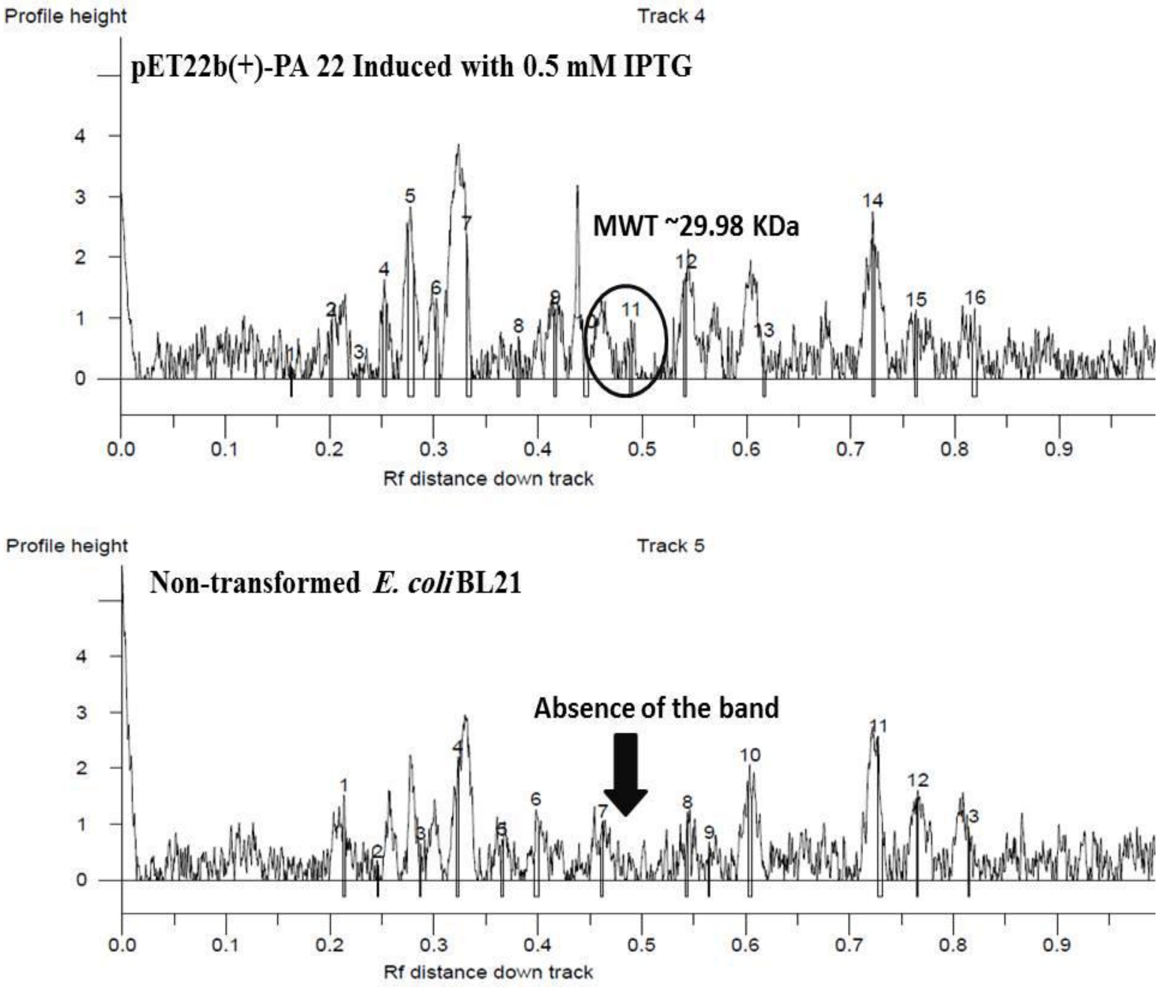

Fig. S11 Rf distance down track plot for electrophoresed protein retrieved from 0.5 mM IPTG induced pET22b(+)-PA22 transformed *E. coli* BL21 (DE3) and non-transformed *E. coli* BL21 (DE3).

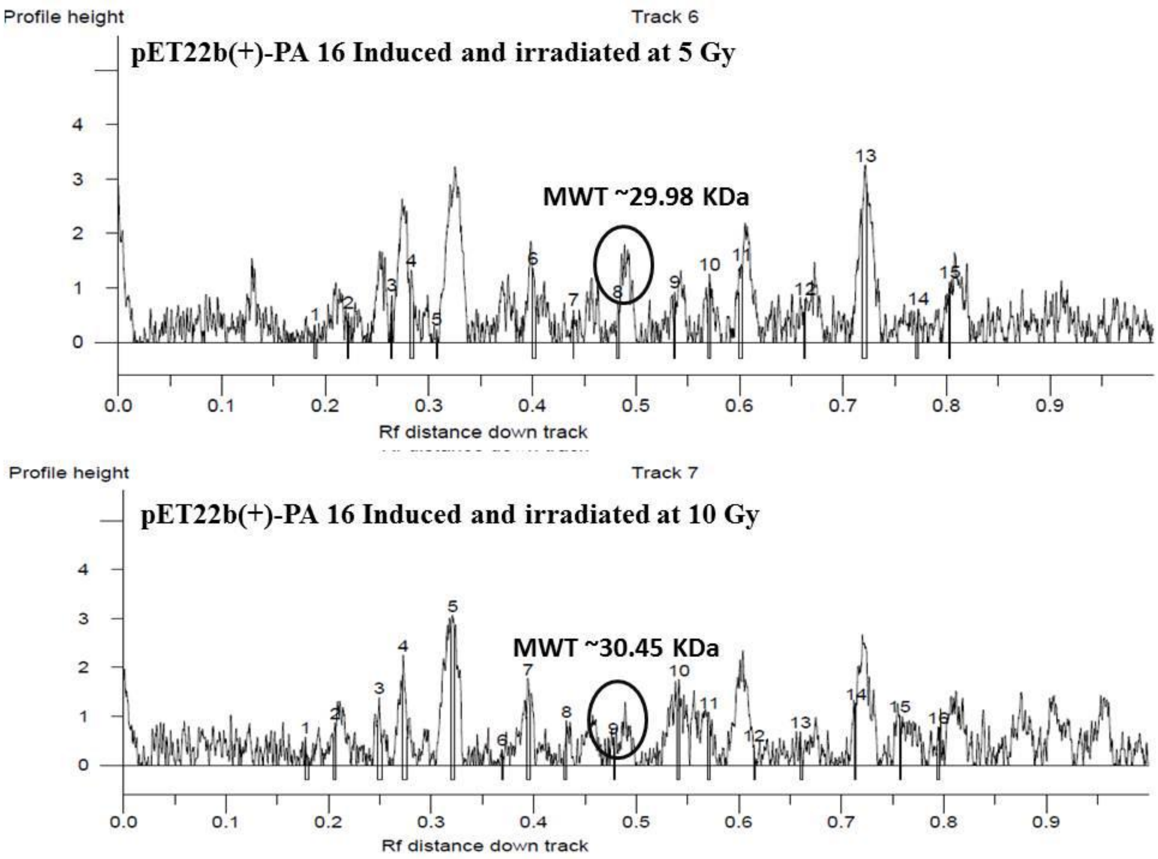

Fig. S12 Rf distance down track plot for electrophoresed protein retrieved from 5 Gy irradiated pET22b(+)-PA16 transformed *E. coli* BL21 (DE3) and 10 Gy irradiated pET22b(+)-PA16 transformed *E. coli* BL21 (DE3) induced by 0.5 mM IPTG.

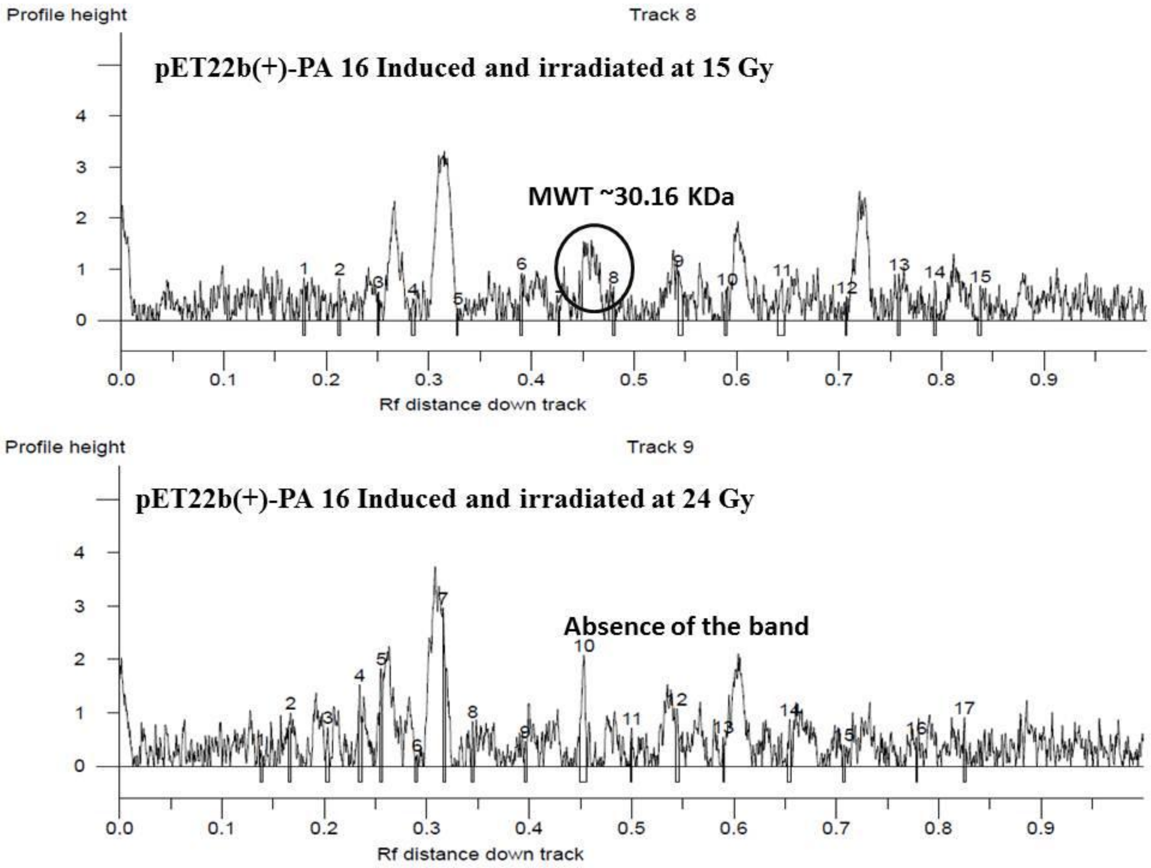

Fig. S13 Rf distance down track plot for electrophoresed protein retrieved from 0.5 mM IPTG induced pET22b(+)-PA16 transformed *E. coli* BL21 (DE3) irradiated at 15Gy and 24 Gy.

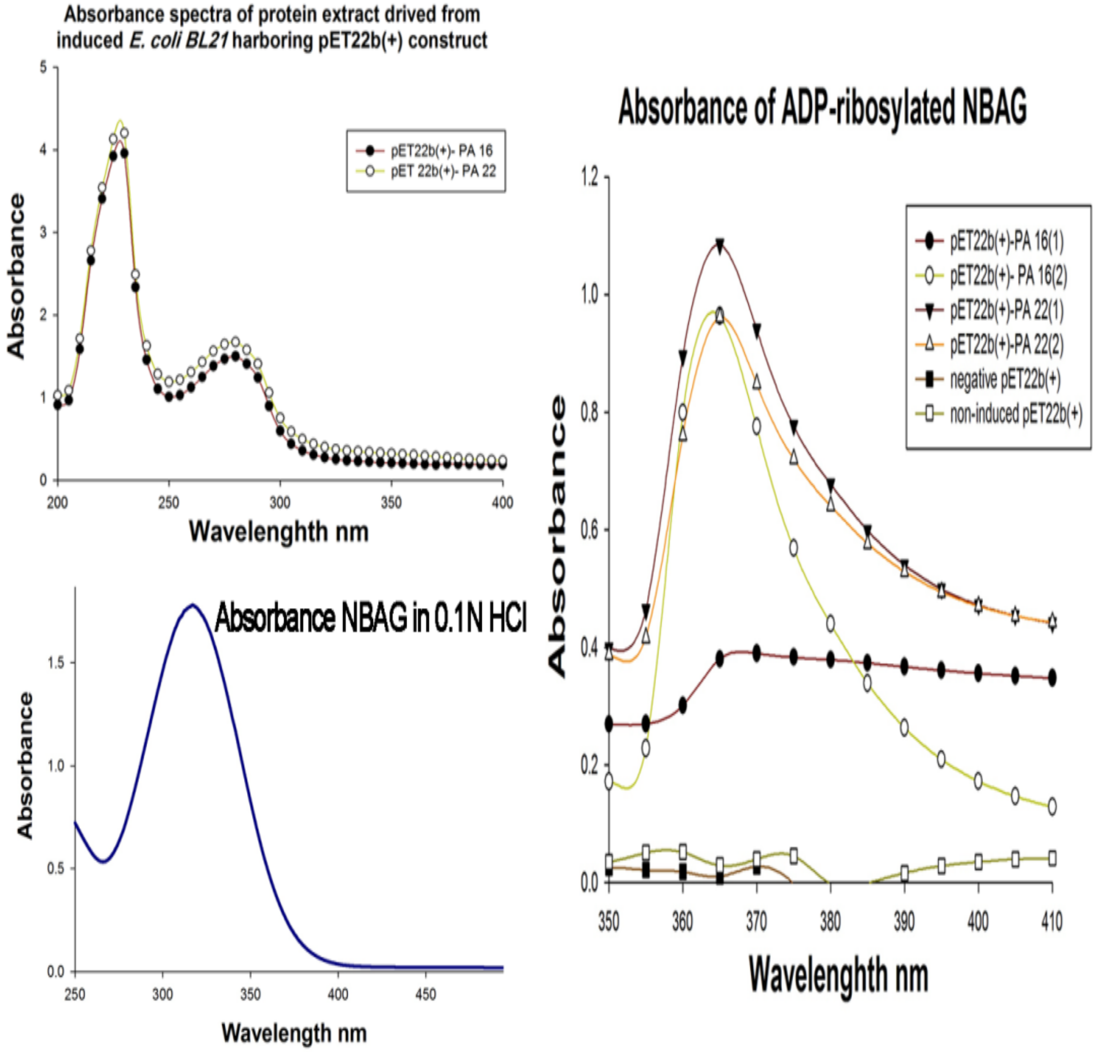

Fig. S14 Absorbance maxima of NBAG post exposure to PE24 moiety retrieved from pET22b(+)-PA16 and pET22b(+)-PA22 transformed *E. coli* BL21(DE3).

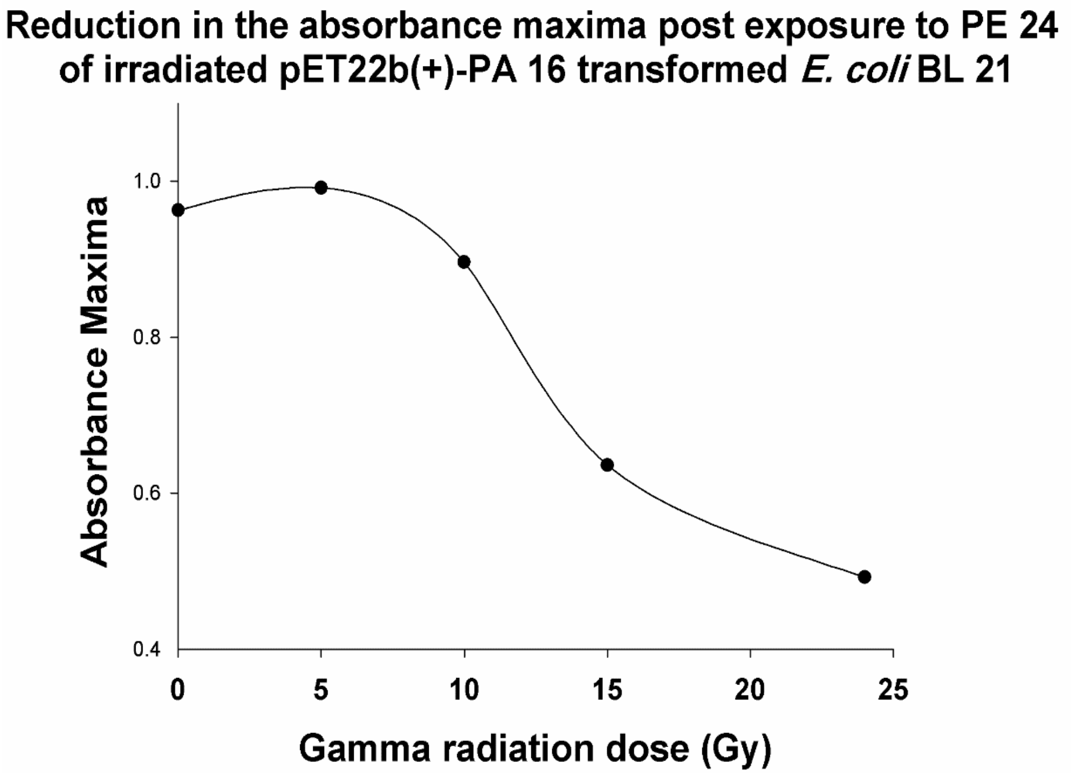

Fig. S15 Reduction in the absorbance maxima post exposure of NBAG reaction tubes to PE 24 extracts retrieved from irradiated pET22b(+)-PA16 *E. coli* BL21(DE3).

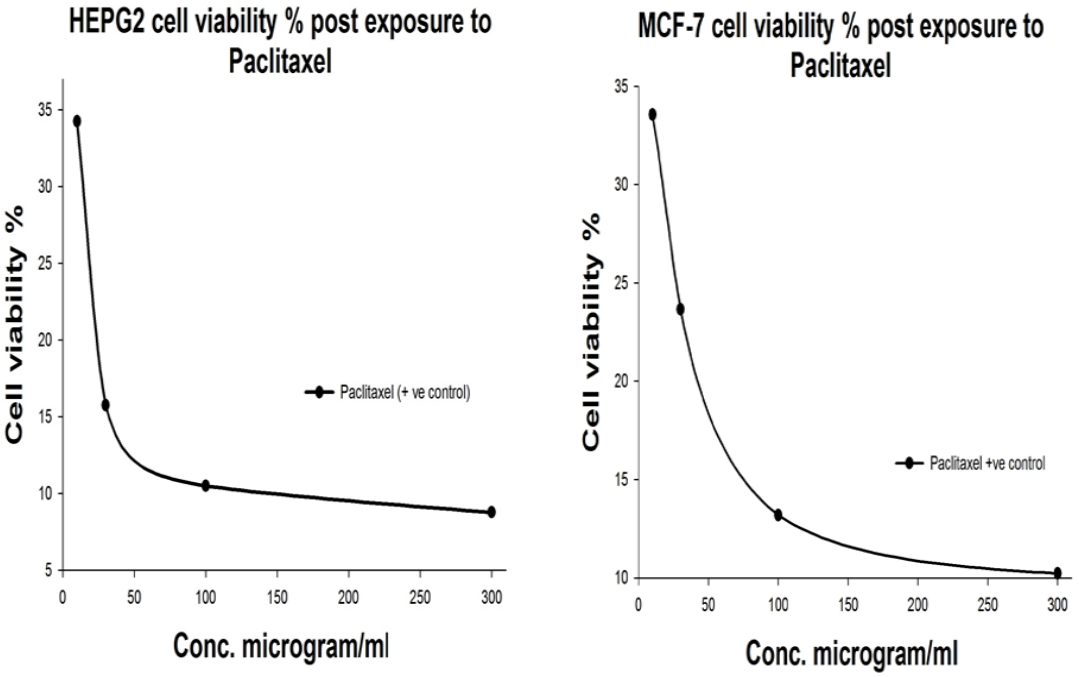

Fig. S16 Plot of cell viability % versus Conc. (μg/ml) of HEPG2 and MCF-7 cells post exposure to +ve control paclitaxel.

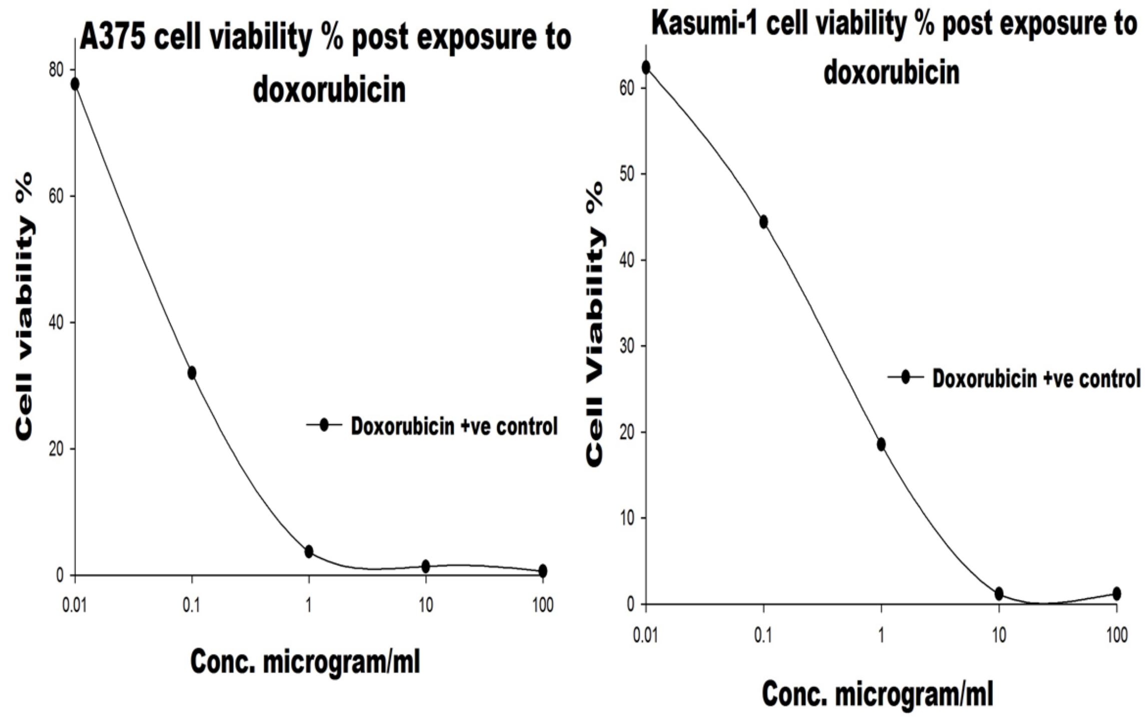

Fig. S17 Plot of cell viability % versus Conc. (μg/ml) of Kasumi-1 and A375 cells post exposure to +ve control doxorubicin.

Tables

Table S1. Percentage of water to methanol in relationship with time for gradient elution of samples

Time (min.)	Water %	Methanol %
0	95	5
3	95	5
10	50	50
20	20	80
25	10	90
27	10	90
29	95	5
32	95	5

Table S2. Raw volume and raw volume % of PE24 moiety at 0.5 mM IPTG and post exposure to low doses gamma radiation

Track no.	Lane	Height	Molecular weight (MWT KDa)	Raw Volume	Raw volume percentage %
3 (pET22b(+)-PA16 induced by 0.5 mM IPTG (unirradiated)	8	0.582	30.22	276.43	4.117
4 (pET22b(+)-PA22 induced by 0.5 mM IPTG)	11	0.986	29.9	425.02	3.836
6 (pET22b(+)-PA16 (5 Gy) irradiated)	8	0.742	29.88	318.63	4.063
7 (pET22b(+)-PA16 (10Gy) irradiated)	9	0.567	30.45	107.93	1.182
8 (pET22b(+)-PA16 (15 Gy) irradiated)	8	0.659	30.16	169.87	3.891

Table S3. Absorbance maxima post exposure of NBAG to PE24 expressed by *E. coli* BL21 (DE3) and amount of ADP-ribosylated NBAG formed.

E.coli **transformed with pET22b(+) construct**	**Absorbance Maxima**	**ADP ribosylated NBAG formed after 1 hr. of incubation (μmol/ml/h)**	**Percentage of ADP ribosylated NABG%**	**Enzymatic activity of PE24 moiety per h. (U/h)**
pET22b(+)-PA16 (1)	0.413	0.206	0.002	0.2
pET22b(+)-PA16 (2)	0.963	0.482	0.00482	0.48
pET22b(+)-PA22 (1)	1.085	0.542	0.00542	0.54
pET22b(+)-PA22 (2)	0.961	0.480	0.0048	0.48

Absorbance maxima; highest absorbance in UV absorbance spectrum: Expected ADP ribosylated NBAG formed = (absorbance maxima x (5x10^{-2}))/0.1: Percentage of ADP ribosylated NBAG% = (conc. of ADP ribosylated NBAG/ conc. of NBAG) x100: Enzymatic activity of PE24 moiety per hour (U/h) = ADP ribosylated NBAG formed per hour (μmol/ml/h).

Table S4. Reduction in ADP-ribosylated products formed by PE24 moiety post-exposure of pET22b(+)-PA16 transformed *E. coli* BL21 (DE3) to low doses gamma irradiation

**Radiation dosage**	**Retention time at 301 nm**	**Peak Area**	**ADP-ribosylated product formed in (M) over 3 h**	**ADP-ribosylated formed per hour (µmol/ml/h)**	**Enzymatic activity of PE24 moiety per hour (U/h)**	**Percent of ADP ribosylated products formed in 3 h. %**	**Reduction in ADP ribosylated NBAG post irradiation in 3 h.**	**Percent reduction in ADP ribosylated NBAG post irradiation %**
				5 Gy				
NBAG Peak	14.001	9427891	0.01					
New peaks	19.11	178592	$1.8*10^{-4}$					
	20.778	40558	$4.3*10^{-5}$					
	21.768	41752	$4.4*10^{-5}$					
	Total		0.00027	0.09	0.09	2.767	16.2	85.3
				10 Gy				
NBAG Peak	14.116	7789280	0.01					
New peaks	19.1	103120	$1.3*10^{-4}$					
	20.77	26972	$3.4*10^{-5}$					
	21.759	27580	$3.5*10^{-5}$					
	Total		0.0002	0.067	0.067	2.024	16.9	89.3
				15 Gy				
NBAG Peak	14.924	4827573	0.01					
New Peaks	19.111	4383	$9.1*10^{-6}$					
	21.369	14157	$2.93*10^{-5}$					
	Total		$3.5*10^{-5}$	0.012	0.012	0.384	18.5	97.9
				24 Gy				
NBAG Peak	14.907	9924125	0.01					
New Peaks	19.118	25970	$2.61*10^{-5}$					
	20.786	6705	$1.77*10^{-8}$					
	Total		$2.61*10^{-5}$	0.0087	0.0087	0.261	18.6	98.6

(M): conc. of NBAG in mole; Gy: Gray; ADP ribosylated product formed: Area of New peak/Area of NBAG peak* Conc. of NBAG; ADP-ribosylated formed per hour (µmol/ml/h) = Total amount of ADP-ribosylated NBAG formed in 3 hs (µmol/ml)/ 3; Enzymatic activity of PE24 moiety per h (U/h) = ADP-ribosylated formed per hour (µmol/ml/h); Percent of ADP ribosylated product formed after 3 h : Total Conc. of ADP ribosylated product formed/Conc. of NBAG *100; Reduction in ADP ribosylated product post irradiation (M): Percent of ADP ribosylated NBAG form pEt22b/PA 16 formed in 3 h – Percent ADP ribosylated NBAG post irradiation dosages formed in 3 h; Percent reduction in ADP ribosylated NBAG post irradiation: Reduction in ADP ribosylated product /Percent of ADP ribosylated products formed form pET22b/PA 16 *100

Table S5. Absorbance maxima and percent reduction in ADP-ribosylated NBAG formed of PE24 moiety post-exposure of transformed *E. coli* BL21 (DE3) to low doses gamma radiation and different metal salts

**Gamma radiation dose (Gy)**	**Absorbance Maxima**	**The expected amount of ADP-ribosylated NBAG formed per hour (µmol/ml/h)**	**Enzymatic activity of PE24 moiety per hour (U/h)**	**Reduction in ADP-ribosylated NBAG**	**Percent reduction in the amount of ADP ribosylated NBAG****
		pET22b(+)-PA 16			
un-irradiated	0.963	0.482	0.482		
5Gy	0.99	0.50	0.50	0	0
10Gy	0.90	0.45	0.45	-0.03	-7.01
15Gy	0.64	0.32	0.32	-0.16	-34.02
24Gy	0.49	0.25	0.25	-0.24	-48.92
		pET22b(+)-PA 22			
un-irradiated	1.085	0.542	0.542		
5Gy	0.85	0.42	0.42	-0.12	-21.83
10Gy	0.68	0.34	0.34	-0.20	-37.18
		pET22b(+)-PA 16 after the addition of different metal salts			
10 mM NaCl	-1.02025	-			
10 mM $ZnCl_2$	-0.86343	-			
10 mM $MgCl_2$	-0.70461	-			
10 mM $MgSO_4$	-0.36343	-			

Absorbance maxima; highest absorbance in UV absorbance spectrum: Expected ADP ribosylated NBAG formed = (absorbance maxima x ($5x10^{-2}$))/0.1: Enzymatic activity of PE24 moiety per hour (U/h) = total amount of ADP-ribosylated NBAG formed per hour (µmol/ml/h):Reduction in the amount of ADP-ribosylated NBAG= conc. of ADP ribosylated NBAG in irradiated samples – conc. of ADP ribosylated NBAG in untreated samples: Percent reduction in the amount of ADP ribosylated NBAG = (reduction in conc. of ADP ribosylated NBAG/conc. of ADP-ribosylated NBAG in untreated samples) x 100

Table S6. Reduction in ADP-ribosylated products formed by PE24 moiety (pET22b(+)-PA16) following the addition of different metal salts to NBAG reaction tubes.

Peaks	Retention time at 301 nm	Peak Area	ADP-ribosylated product formed in (M) after 3 h	ADP-ribosylated NBAG formed per hour (µmol/ml/h)	Enzymatic activity of PE24 per hour (U/h)	Percent of ADP ribosylated products formed	Reduction in ADP ribosylated NBAG post addition of metal salt in 3 h	Percent reduction in ADP ribosylated NBAG post addition of metal salt %
				10 mM NaCl				
NBAG Peak	19.527	2512350	0.01					
New peaks	21.401	35007	$1.3*10^{-4}$					
	22.789	13495	$5.3*10^{-5}$					
	25.075	19165	$7.6*10^{-5}$					
Total			$2.69*10^{-4}$	0.09	0.09	2.69	16.2	85.7
				10 mM $ZnCl_2$				
NBAG Peak	19.585	2481410	0.01					
New Peaks	21.476	34946	$1.4*10^{-4}$					
	22.816	13271	$5.3*10^{-5}$					
	25.691	19312	$7.7*10^{-5}$					
	30.079	16561	$6.6*10^{-5}$					
Total			$3.3*10^{-4}$	0.11	0.11	3.38	15.5	82.09
				10 mM $MgCl_2$				
NBAG Peak	19.619	2370530	0.01					
New Peaks	21.508	36016	$1.5*10^{-4}$					
	22.831	13195	$5.56*10^{-5}$					
	26.031	18320	$7.7*10^{-5}$					
	30.094	18198	$7.6*10^{-5}$					
Total			$3.6*10^{-4}$	0.12	0.12	3.61	15.3	80.8
				10 mM $MgSO_4$				
NBAG Peaks	19.631	2502770	0.01					
New Peaks	21.521	33769	$1.3*10^{-4}$					
	22.822	13623	$5.4*10^{-5}$					
	26.321	20442	$8.1*10^{-5}$					
Total			$2.7*10^{-4}$	0.09	0.09	2.71	16.2	85.6

(M): conc. of NBAG in mole; ADP ribosylated product formed: Area of New peak/Area of NBAG peak* Conc. of NBAG; Percent of ADP ribosylated product formed: Total Conc. of ADP ribosylated product formed/Conc. of NBAG *100; ADP-ribosylated formed per hour (µmol/ml/h) = Total amount of ADP-ribosylated NBAG formed in 3 hs (µmol/ml)/ 3; Enzymatic activity of PE24 moiety per h (U/h) = ADP-ribosylated formed per hour (µmol/ml/h); Reduction in ADP ribosylated product post exposure to metal salt= Percent ADP ribosylated NBAG

formed in pEt22b/PA 16 after 3 h – Percent ADP ribosylated NBAG formed in 3 h. post exposure to metal salts; Percent reduction in ADP ribosylated product formed post-exposure metal salts = Reduction in total ADP ribosylated NBAG / percent ADP ribosylated NBAG form pET22b/PA 16 (M) *100

Table S7. Percent cell cytotoxicity and viability of PE24 extract retrieved from pET22b(+)-PA16 transformed *E. coli* BL21 (DE3) alone and in combination with paclitaxel HEPG2 cells.

**Paclitaxel (+ve control) conc. µg/ml**	**Cell viability %**	**Cell cytotoxicity%**	
300	8.76	91.23 ± 0.259	
100	10.49	89.50 ± 0.326	
30	15.75	84.24 ± 0.768	
10	34.22	65.77 ± 1.344	
3	39.46	60.54 ± 1.421	
Equation		$y=35.56e^{(-0.0157X)}$	
R^2 value		0.6636	
**pET22b/PA 16 Toxin extract conc. µg/ml**	**Cell viability %**	**Cell cytotoxicity%**	**P value**
300	9.41	90.59 ± 0.174	$7.58*10^{-11}$
100	11.40	88.60 ± 0.517	$1.11*10^{-10}$
30	21.96	78.04 ± 0.341	$2.16*10^{-10}$
10	50.21	49.79 ± 1.089	$6.73*10^{-9}$
3	63.02	36.98 ± 1.566	$6.39*10^{-9}$
Equation		$y= 69.07e^{(-0.033X)}$	
R^2 value		0.9222	
IC50 µg/ml		7.86	
**pET22b/PA 16 Toxin extract conc. µg/ml in combination with 5 µg/ml paclitaxel**	**Cell viability %**	**Cell cytotoxicity%**	**P value**
300	10.13	89.87 ± 0.201	$9.96*10^{-11}$
100	11.71	88.29 ± 0.653	$1.25*10^{-10}$
30	22.28	77.72 ± 1.53	$1.08*10^{-9}$
10	40.48	59.52 ± 0.576	$2.88*10^{-9}$
3	60.92	39.08 ± 0.672	$2.051*10^{-9}$
Equation		$y=63.21e^{(-0.0335X)}$	
R^2 value		0.8758	
IC50 µg/ml		6.48	
Reduction in IC50 value		1.38	
Percent reduction in IC50 %		17.5%	

Reduction in IC50: pET22b(+)/PA 16 toxin extract IC50- pET22b(+)/PA 16 toxin extract in combination with 5 µg/ml paclitaxel IC50; Percent reduction in IC50%: Reduction in IC50/ pET22b(+)/PA 16 toxin extract IC50 *100

Table S8. Percent cell cytotoxicity and viability of PE 24 extract retrieved from pET22b(+)-PA16 transformed *E. coli* BL21 (DE3) alone and in combination with paclitaxel on MCF-7 cells

**Paclitaxel (+ve control) conc. µg/ml**	**Cell viability %**	**Cell cytotoxicity%**	
300	10.21	89.78 ± 0.565	
100	13.18	86.81 ± 0.327	
30	23.65	76.35 ± 3.28	
10	33.54	66.45 ± 1.38	
3	29.56	70.44 ± 0.76	
Equation		$y=31.63e^{(-0.0061X)}$	
R^2 value		0.8113	
**pET22b/PA 16 Toxin extract conc. µg/ml**	**Cell viability %**	**Cell cytotoxicity%**	**P value**
300	12.72	87.27 ± 0.663	$3.64*10^{-11}$
100	13.89	86.1 ± 0.280	$7.45*10^{-12}$
30	28.86	71.13 ± 1.98	$1.05*10^{-8}$
10	40.75	59.24 ± 0.687	$1.68*10^{-9}$
3	67.93	32.06 ± 0.92	$4.86*10^{-10}$
Equation		$y=64.78e^{(-0.0254X)}$	
R^2 value		0.8105	
IC50 µg/ml		9.96	
**pET22b/PA 16 Toxin extract conc. µg/ml in combination with 5 µg/ml paclitaxel**	**Cell viability %**	**Cell cytotoxicity%**	**P value**
300	9.89	90.10 ± 0.297	$3.25*10^{-11}$
100	12.70	87.29 ± 0.301	$1.13*10^{-11}$
30	23.44	76.56 ± 1.58	$3.59*10^{-9}$
10	40.91	59.08 ± 0.89	$2.13*10^{-9}$
3	58.60	41.39 ± 1.09	$4.23*10^{-10}$
Equation		$Y= 58.83e^{(-0.026X)}$	
R^2 value		0.8712	
IC50 µg/ml		5.86	
Reduction in IC50		4.1	
Percent reduction in IC50		41.1%	

Reduction in IC50: pET22b(+)/PA 16 toxin extract IC50- pET22b(+)/PA 16 toxin extract in combination with 5 µg/ml paclitaxel IC50; Percent reduction in IC50%: Reduction in IC50/ pET22b(+)/PA 16 toxin extract IC50 *100

Table S9. Percent cell cytotoxicity and viability of PE24 extract retrieved from pET22b(+)-PA16 transformed *E. coli* BL21 (DE3) on A375: Human Melanoma cell lines

**Doxorubicin (+ve control) conc. µg/ml**	**Cell viability %**	**Cell cytotoxicity%**	
0.01	77.68 ± 1.29	22.31	
0.1	31.99 ± 0.617	68	
1	3.73 ± 2.66	96.26	
10	1.35 ± 0.712	98.64	
100	0.63 ± 0.841	99.36	
Equation		$y=85.72e^{(-9.856\ X)}$	
R^2 value		0.9963	
**pET22b/PA 16 Toxin extract conc. µg/ml**	**Cell viability %**	**Cell cytotoxicity%**	**P value**
0.01	102.44 ± 0.568	0	$6.35*10^{-7}$
0.1	99.71 ± 0.0751	0.284	$9.52*10^{-11}$
1	97.47 ± 0.93	2.52	$3.54*10^{-9}$
10	39.45 ± 1.805	60.54	$1.026*10^{-9}$
100	2.59 ± 1.73	97.40	$4.41*10^{-10}$
Equation		$Y=103.1148e^{(-0.0941X)}$	
R^2 value		0.9967	
IC50 µg/ml		7.2	

Table S10. Percent cell cytotoxicity and viability of PE 24 extract retrieved from pET22b(+)-PA16 transformed *E. coli* BL21 (DE3) on OEC and Kasumi-1 cell lines

**Oral Epithelial cells**			
pET22b+/PA-16 toxin extract	Cell viability %	Cell cytotoxicity %	
10 μg/ml	90.93	9.064±0.813	
100 μg/ml	5.66	94.33±0.118	
Kasumi-1: acute monocytic leukemia (AML)			
Doxorubicin μg/ml (+ve control)	Cell viability%	Cell cytotoxicity%	
0.01	63.89	37.63±1.35	
0.1	45.76	55.56±1.54	
1	20.7	81.45±2.49	
10	1.2	98.81±0.083	
100	0.098	99.88±0.092	
Equation		$y=57.73e^{(-1.238X)}$	
R^2 Value		0.9736	
pET22b/PA 16 Toxin extract conc. μg/ml	Cell viability%	Cell cytotoxicity%	P value
0.01	87.86	12.14±0.869	$3.79*10^{-8}$
0.1	83.14	16.86±2.07	$3.42*10^{-8}$
1	82.56	17.44±2.24	$1.14*10^{-8}$
10	39.67	60.33±1.39	$6.50*10^{-11}$
100	3.13	96.87±1.52	$4.33*10^{-11}$
Equation		$Y=86.81e^{(-0.0773X)}$	
R^2 Value		0.9977	
IC50 μg/ml		6.98	

Table S11. Cytotoxicity of PE24 moiety on gamma irradiated on HEPG2 cells

pET22b/PA 16 PE24 toxin extract conc. µg/ml	Cell viability %	Cell cytotoxicity%	P value
		At 5 Gy	
300	15.35	83.99 ± 0.912	0.00012
100	27.42	72.57 ± 0.456	$1.07*10^{-5}$
30	40.14	59.85 ± 0.182	$1.31*10^{-6}$
10	50.40	49.59 ± 0.821	$6.94*10^{-7}$
3	66.98	33.01 ± 1.27	$3.032*10^{-7}$
Equation		$Y= 58.09\ e^{(-0.0067X)}$	
R^2 value		0.9242	
IC50 µg/ml		10	
Increase in IC50		2.14	
Percent increase in IC50		27.2%	
		At 24 Gy	
300	12.08	87.91 ± 0.292	0.0003
100	24.84	75.15 ± 0.638	$1.29*10^{-5}$
30	36.59	63.40 ± 1.00	$3.06*10^{-5}$
10	46.78	53.21 ± 0.821	$9.27*10^{-5}$
3	62.85	37.14 ± 0.365	$2.072*10^{-7}$
Equation		$Y= 55.0435\ e^{(-0.0078X)}$	
R^2 value		0.9345	
IC50 µg/ml		12.28	
Increase in IC50		4.42	
Percent increase in IC50		56	

Increase in IC50: PE24 IC50 on gamma irradiated HEPG2 - PE24 IC50 on un-irradiated HEPG2;
Percent increase in IC50%: Increase in IC50/ PE24 IC50 *100

Table S12. Cytotoxicity of PE24 coupled with low doses of gamma radiation on HEPG2 cells

pet22b+/PA-16 toxin extract conc. (μg/ml)	Cell viability%	Cell cytotoxicity%	P value
	**HEPG2 cells Un-irradiated**		
0.01	99.21	0.47±1.28	0.453
0.1	95.09	4.90±0.449	0.0004
1	93.71	6.28±0.937	0.0007
10	45.12	54.87±2.42	$2.92*10^{-8}$
100	1.47	98.52±0.785	$6.46*10^{-9}$
Equation		$Y=98.60e^{(-0.0772X)}$	
R^2 value		0.9970	
IC50		8.66 μg/ml	
	Irradiated at 5 Gy		
0.01	99.59	0.026±0.632	0.675
0.1	95.98	4.01±0.696	0.00016
1	93.87	6.129±0.778	0.00012
10	47.74	52.25±1.95	$4.36*10^{-8}$
100	3.29	96.70±0.498	$2.02*10^{-12}$
Equation		$Y=95.95e^{(-0.0722X)}$	
R^2 value		0.9973	
IC50		9.38 μg/ml	
Percentage increase in IC50 post-irradiation		8.3%	
	Irradiated at 24 Gy		
0.01	99.69	0.304±0.24	0.537
0.1	97.05	2.94±0.741	0.00026
1	96.21	3.78±0.531	0.00014
10	53.53	46.46±1.52	$3.59*10^{-8}$
100	2.12	97.87±0.64	$2.91*10^{-12}$
Equation		$Y=99.73e^{(-0.0614X)}$	
R^2 value		0.9981	
IC50		10.94 μg/ml	
Percentage increase in IC50 post-irradiation		26.32%	

Increase in IC50: PE24 IC50 along with gamma radiation dose- PE24 IC50 on un-irradiated HEPG2; Percent increase in IC50%: Increase in IC50/ PE24 IC50 un-irradiated*100
